# Dual targeting of MTOR as a novel therapeutic approach for high-risk B-cell acute lymphoblastic leukemia

**DOI:** 10.1038/s41375-021-01132-5

**Published:** 2021-02-02

**Authors:** Zheng Ge, Chunhua Song, Yali Ding, Bi-Hua Tan, Dhimant Desai, Arati Sharma, Raghavendra Gowda, Feng Yue, Suming Huang, Vladimir Spiegelman, Jonathon L. Payne, Mark E. Reeves, Soumya Iyer, Pavan Kumar Dhanyamraju, Yuka Imamura, Daniel Bogush, Yevgeniya Bamme, Yiping Yang, Mario Soliman, Shriya Kane, Elanora Dovat, Joseph Schramm, Tommy Hu, Mary McGrath, Zissis C. Chroneos, Kimberly J. Payne, Chandrika Gowda, Sinisa Dovat

**Affiliations:** 1 Pennsylvania State University College of Medicine, Hershey, PA 17033 USA; 2 Zhongda Hospital, Medical School of Southeast University Nanjing, 210009 Nanjing, China; 3 Ohio State University College of Medicine, Columbus, OH 43210 USA; 4 Loma Linda University College of Medicine, Loma Linda, CA 92350 USA

**Keywords:** Targeted therapies, Cell signalling

## Abstract

Children of Hispanic/Latino ancestry have increased incidence of high-risk B-cell acute lymphoblastic leukemia (HR B-ALL) with poor prognosis. This leukemia is characterized by a single-copy deletion of the *IKZF1* (IKAROS) tumor suppressor and increased activation of the PI3K/AKT/mTOR pathway. This identifies mTOR as an attractive therapeutic target in HR B-ALL. Here, we report that IKAROS represses *MTOR* transcription and IKAROS’ ability to repress *MTOR* in leukemia is impaired by oncogenic CK2 kinase. Treatment with the CK2 inhibitor, CX-4945, enhances IKAROS activity as a repressor of *MTOR*, resulting in reduced expression of *MTOR* in HR B-ALL. Thus, we designed a novel therapeutic approach that implements dual targeting of mTOR: direct inhibition of the mTOR protein (with rapamycin), in combination with IKAROS-mediated transcriptional repression of the *MTOR* gene (using the CK2 inhibitor, CX-4945). Combination treatment with rapamycin and CX-4945 shows synergistic therapeutic effects in vitro and in patient-derived xenografts from Hispanic/Latino children with HR B-ALL. These data suggest that such therapy has the potential to reduce the health disparity in HR B-ALL among Hispanic/Latino children. The dual targeting of oncogene transcription, combined with inhibition of the corresponding oncoprotein provides a paradigm for a novel precision medicine approach for treating hematological malignancies.

## Introduction

Children with Hispanic/Latino ancestry have a higher incidence of acute lymphoblastic leukemia (ALL) and increased mortality from this disease [1]. The incidence of a specific subtype of high-risk B-ALL, characterized by an *IGH/CRLF2* translocation, is highly increased in these children [2]. The translocation of the *cytokine receptor-like factor 2* (*CRLF2*) gene results in increased expression of *CRLF2* [3] and increased activation of its downstream signaling pathways [4]. CRLF2 is an upstream regulator of the PI3K/AKT/mTOR pathway and increased *CRLF2* expression leads to upregulation of this pathway [4]. Aberrant activation of the PI3K/AKT/mTOR pathway is frequently detected in high-risk B-ALL and is associated with chemoresistance and poor prognosis [5, 6]. mTOR (mammalian Target of Rapamycin) is a serine/threonine kinase that directly regulates cellular proliferation and metabolism [7, 8]. Increased expression and activation of mTOR are associated with poor outcomes in ALL [9, 10].

The *IKZF1* gene encodes IKAROS, which acts as a tumor suppressor in B-ALL [11–13]. IKAROS regulates gene expression via chromatin remodeling [14–18]. Reduced IKAROS activity due to deletion and/or inactivating mutations often results in high-risk B-ALL, which is associated with resistance to chemotherapy [19–31]. We have shown that the deletion of one *IKZF1* allele is highly increased in B-ALL of Hispanic/Latino children. Further, we found that the combination of *IKZF1* deletion and *CRLF2* translocation is 15 times more common in this group than in the non-Hispanic/Latino pediatric patient population^COMPANION PAPER^. Subtypes of B-ALL with *CRLF2* translocation and *IKZF1* deletion frequently give rise to Ph-like ALL [32]. Treatment of Ph-like ALL is challenging, thus B-ALL with *CRLF2* overexpression and/or *IKZF1* deletion are often resistant to conventional treatment, and have an increased incidence of relapse and poor prognosis [31–33]. Novel targeted treatment is essential to reduce health disparities in Hispanic/Latino children with B-ALL.

Here, we report that IKAROS represses transcription of the *MTOR* gene via chromatin remodeling. In high-risk B-ALL, IKAROS’s ability to regulate *MTOR* expression is abolished due to deletion of one *IKZF1* allele and phosphorylation of the IKAROS protein by oncogenic Casein Kinase II (CK2). Inhibition of CK2 restores IKAROS-mediated transcriptional repression of *MTOR* in B-ALL. Combination treatment with CX-4945 and the direct mTOR inhibitor, rapamycin, shows synergistic therapeutic effects in vitro and in preclinical models of high-risk B-ALL from Hispanic/Latino children. These data lay the groundwork for clinical testing of combination therapy that targets both *MTOR* gene expression (via restoration of IKAROS activity by CK2 inhibition), as well as direct inhibition of the mTOR protein (with rapamycin) in high-risk B-ALL. Such therapy has the potential to reduce the health disparity experienced by Hispanic/Latino children with high-risk B-ALL.

## Materials, subjects and methods

### Cell culture and reagents

The Nalm6, 697 (EU-3), and HEK-293T (293T) cell lines have been described previously [34, 35]. Primary human B-ALL cells were cultured as described previously [36, 37].

### High-risk B-ALL patient-derived xenograft models

2 × 10^6^ cells per mouse were transplanted intravenously into 4-week-old female NOD.Cg-*Rag1*^*tm1Mom*^ *Il2rg*^*tm1Wjl*^/SzJ (NRG) mice. Following engraftment, mice received vehicle only, CX-4945 (daily *via* gavage at 100 mg/kg/day), rapamycin (4 mg/kg intraperitoneal injection [IP] 5 days/week) or combination treatment with CX-4945 (100 mg/kg/day) and rapamycin (4 mg/kg IP 5 days/week) until death of the first animal in experiment (3–5 weeks). Following the treatment period, total living cells in the bone marrow (BM) and spleen of mice were determined by hemocytometer count, and were used in combination with flow cytometry to calculate leukemia burden as reported previously [37].

ChIP-Seq data are accessible on NCI Gene Expression Omnibus (GEO) under accession numbers GSE58825, GSE44218, and GSE141572

Additional details regarding reagents, animal studies, patient samples, experimental methods, and data accession are found in the online Supplemental Materials.

## Results

### IKAROS represses transcription of *MTOR*

**Fig. 1:**
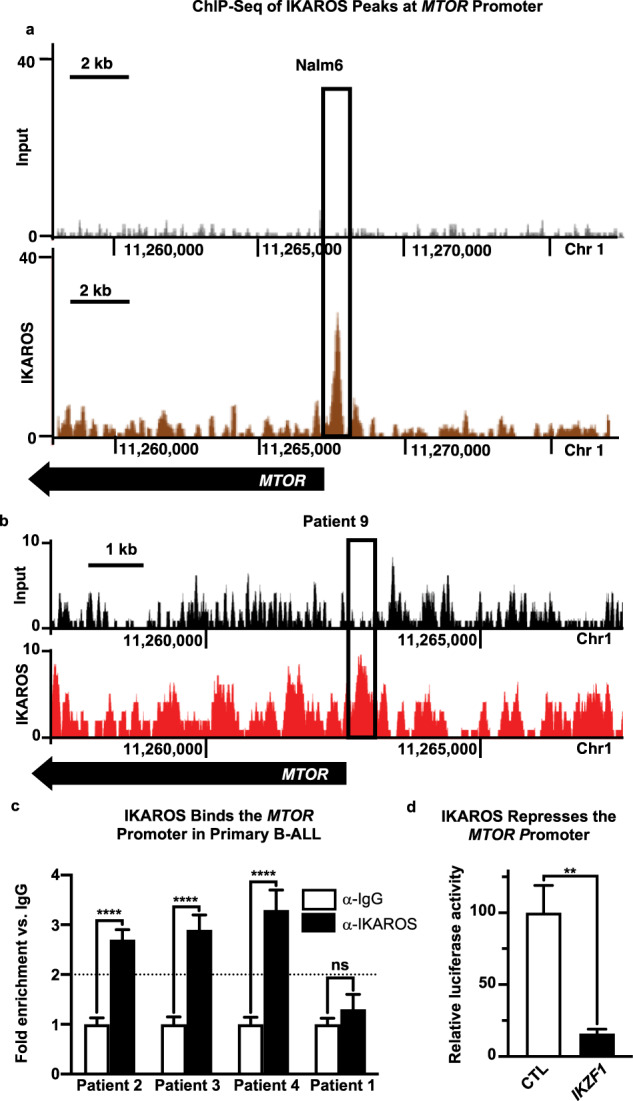
IKAROS binds to the promoter of the *MTOR* gene and suppresses mTOR expression. **a** IKAROS binding sites were identified by ChIP-Seq at the *MTOR* promoter in the (**a**) Nalm6 B-ALL cell lines and in a (**b**) B-ALL patient sample. **c** qChIP data confirming IKAROS binding at the *MTOR* promoter in primary B-ALL cells with wildtype *IKZF1* (Patients 2–4) but not in *IKZF1* haploinsufficiency (Patient 1). **d** Activity of the *MTOR* promoter (−1 kb to +500 bp) was assessed by luciferase reporter assay following transfection with *IKZF1* plasmids or control vector in 293T cells.

Analysis of global DNA binding showed that IKAROS binds to the *MTOR* promoter region in B-ALL cell lines and primary cells from patients (Figs. 1a-b, and S1a-b). IKAROS binding at the *MTOR* promoter was determined by a quantitative chromatin immunoprecipitation (qChIP) assay of primary B-ALL cells (Fig. 1c), and B-ALL cell lines (Fig. S2). IKAROS binding at the *MTOR* promoter was not detected in primary B-ALL with a deletion of one *IKZF1* allele, Fig. 1c, (Patient 1) or in 293T cells, which do not express IKAROS (Fig. S2).

The direct effect of IKAROS binding at the *MTOR* promoter was studied using a transient co-transfection assay with the *MTOR* promoter, that spans −1 kb to +500 bp relative to the *MTOR* transcription start site (TSS). Co-transfection of *IKZF1* resulted in decreased luciferase activity, suggesting that IKAROS binding to the *MTOR* promoter represses transcription (Fig. 1d).

### IKAROS represses transcription of *MTOR* in B-ALL via chromatin remodeling

**Fig. 2:**
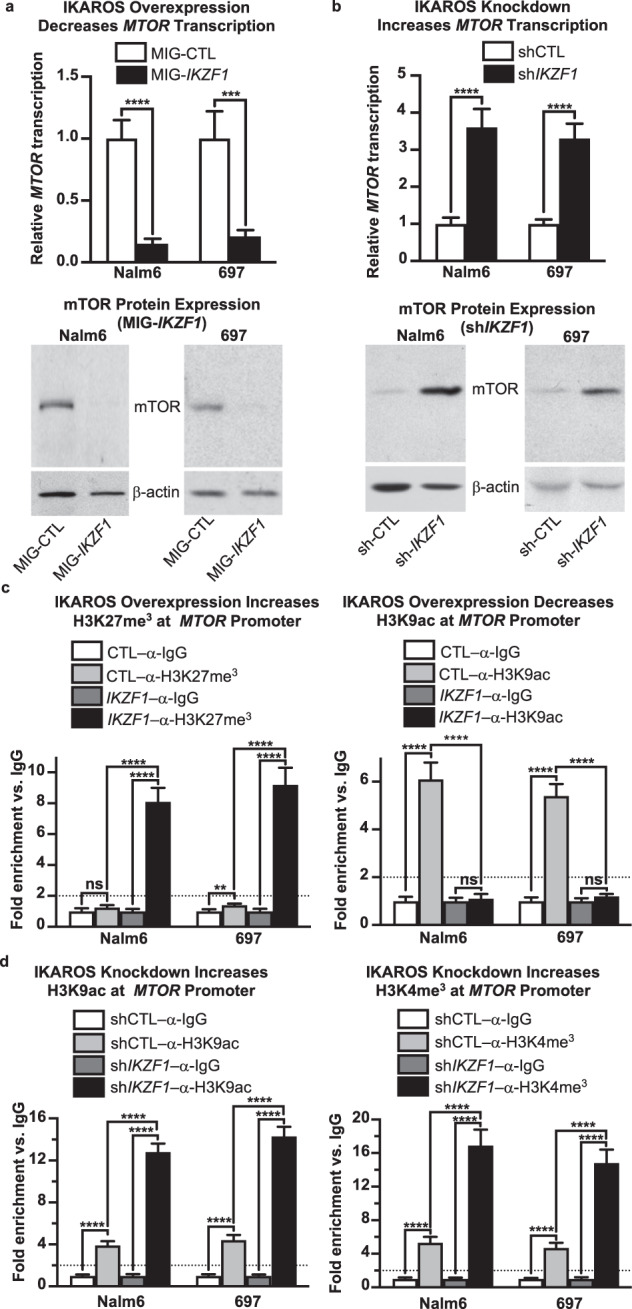
IKAROS represses transcription of *MTOR* in B-ALL via chromatin remodeling. **a** Nalm6 and 697 B-ALL cell lines were transduced to express *IKZF1* (MIG-*IKZF1*) or with empty vector (MIG-CTL). Relative expression of *MTOR* was assessed by qRT-PCR (top panel) and by Western blot (bottom panel). **b** Nalm6 and 697 B-ALL cell lines were treated with *IKZF1* shRNA (sh*IKZF1)* or control shRNA (shCTL). The relative expression of *MTOR* was assessed by qRT-PCR (top panel) and by Western blot (bottom panel). **c** qChIP data showing the presence of H3K27me^3^ and H3K9ac marks at the *MTOR* promoter in Nalm6 and 697 B-ALL cells with IKAROS overexpression. **d** qChIP data showing H3K9ac and H3K4me^3^ marks at the *MTOR* promoter in Nalm6 and 697 B-ALL cells with *IKZF1* shRNA knockdown. Cells were treated for 3 days for the experiments in (**a**–**d**); graphed data are the mean ± SD of combined values from three independent experiments. ***p < 0.001, ****p < 0.0001.

We tested the effect of increased IKAROS expression on *MTOR* transcription in Nalm6 and 697 B-ALL cells. Increased IKAROS expression following retroviral transduction resulted in increased IKAROS occupancy at the *MTOR* promoter (Fig. S3a-b), and reduced expression of *MTOR* as measured by qRT-PCR and Western blot (Fig. 2a).

Targeting IKAROS with shRNA in Nalm6 and 697 cell lines resulted in a loss of IKAROS binding at the *MTOR* promoter (Fig. S3c-d), and increased expression of *MTOR*, as measured by qRT-PCR and Western blot, (Fig. 2b).

Overexpression of IKAROS resulted in increased H3K27me^3^ and a loss of H3K9ac (Fig. 2c) at the *MTOR* promoter. Targeting *IKZF1* with shRNA resulted in increased H3K9ac and H3K4me^3^ at the *MTOR* promoter (Fig. 2d).

Taken together, these data provide evidence that IKAROS acts as a transcriptional repressor of *MTOR* in B-ALL via formation of repressive chromatin at the *MTOR* promoter.

### CK2 inhibits IKAROS-mediated repression of *MTOR*

**Fig. 3:**
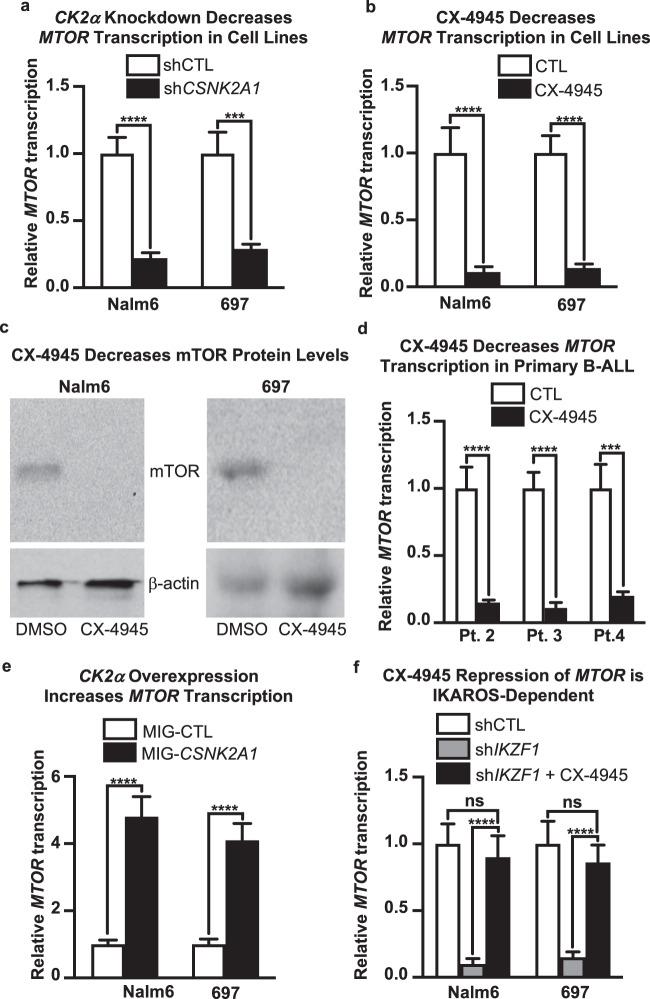
CK2 inhibits IKAROS-mediated repression of *MTOR*. **a**–**b** Effect of CK2α knockdown on mRNA levels of (**a**) *MTOR*. Effect of pharmacological inhibition of CK2 (with CX-4945) on *MTOR* expression in (**b**–**c**) cell lines and in (**d**) patients 2–4. **e** Effect of CK2α overexpression (MIG-*CSNK2A1*) and vector only control (MIG-CTL) on *MTOR* expression. **f** Effect of IKAROS knockdown (sh*IKZF1*) or scramble shRNA control (shCTL) on changes in *MTOR* gene expression induced by CK2 inhibition with CX-4945. Cells were treated with 5 μM CX-4945 for 2 days in (**b**–**d**) and (**f**). Patients 2 and 3 are Hispanic/Latino, patient 4 is non-Hispanic/Latino. Graphed data are the mean ± SD of combined values from three independent experiments. ***p < 0.001, ****p < 0.0001.

In leukemia, direct phosphorylation by CK2 reduces IKAROS DNA-binding affinity and its activity as a transcriptional regulator [38, 39]. We tested whether CK2 regulates IKAROS’ ability to control *MTOR* transcription in B-ALL. Molecular inhibition of *CK2* with shRNA targeting the catalytic subunit of the CK2 holoenzyme, *CK2α*, (Fig. S4a), resulted in repression of the *MTOR* gene (Fig. 3a), along with increased binding of IKAROS at the *MTOR* promoter (Fig. S4b). A similar effect was achieved with pharmacological inhibition of CK2 with specific inhibitors, CX-4945 (Fig. 3b–c, Fig. S5-S6a) and TBB (Fig. S7a-b) in B-ALL cells. Treatment with CX-4945 resulted in increased H3K27me^3^ and a loss of H3K9ac at the *MTOR* promoter (Fig. S8). Since the PI3K/AKT/mTOR pathway is often upregulated in B-ALL in Hispanic/Latino children [33, 40], we tested the effect of CK2 inhibition on IKAROS’s ability to regulate *MTOR* transcription in primary B-ALL cells from pediatric patients that were Hispanic/Latino (patients 2 and 3), as well as non-Hispanic/Latino (patient 4). CK2 inhibition with CX-4945 increased IKAROS binding at the *MTOR* promoter (Fig. S6b) and severely repressed *MTOR* transcription (Fig. 3d).

Overexpression of *CK2α* in B-ALL cells results in increased transcription of the *MTOR* gene (Fig. 3e), and a loss of IKAROS binding at the *MTOR* promoter (Fig. S9). We tested whether IKAROS is the critical protein through which CK2 regulates *MTOR* expression. Treatment of B-ALL cells with the CK2 inhibitor, CX-4945, along with scrambled shRNA, resulted in reduced transcription of the *MTOR* gene; however, *IKZF1* knockdown with shRNA was able to rescue CX-4945-mediated repression of *MTOR* in both cell lines (Fig. 3f). These data show that IKAROS activity is essential for the repression of the *MTOR* gene following CK2 inhibition and suggest that CK2 inhibitors repress *MTOR* expression by enhancing the function of IKAROS.

Together, these data demonstrate that in B-ALL the expression of *MTOR* is regulated by the CK2-IKAROS signaling axis, and that alteration in activity of CK2 and/or IKAROS results in changes in *MTOR* expression.

### CK2 inhibition restores IKAROS’ ability to regulate *MTOR* expression in high-risk leukemia cells from Hispanic/Latino children

**Fig. 4:**
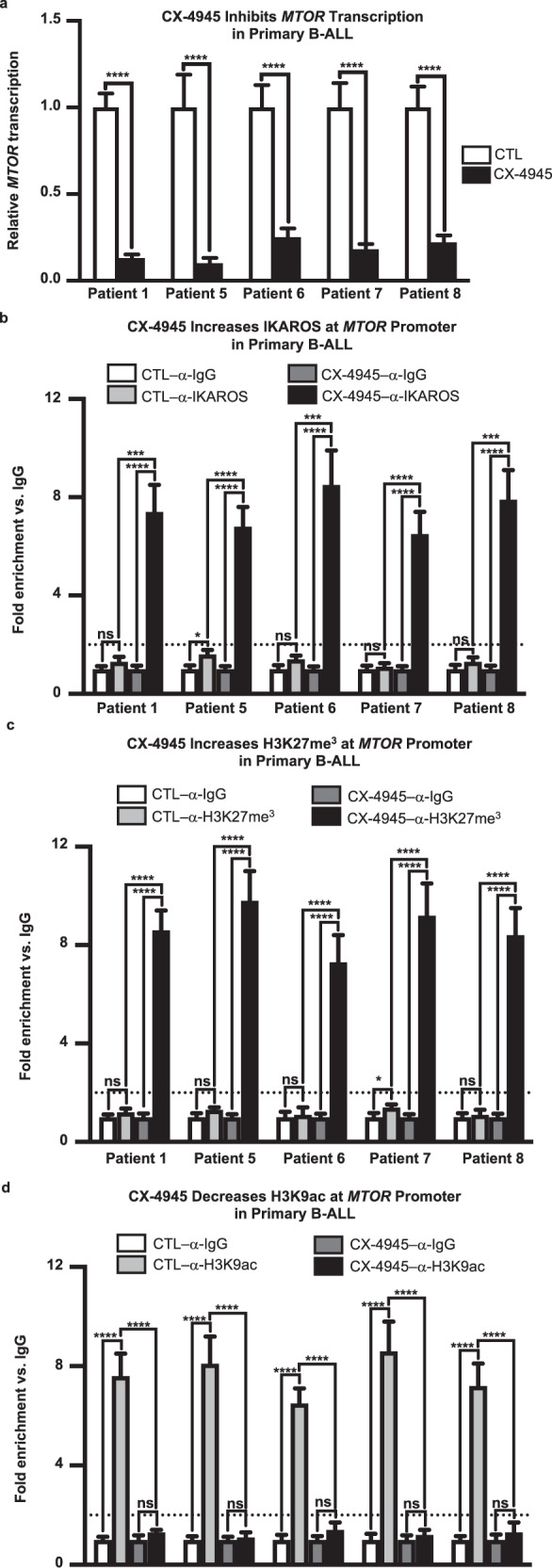
CK2 inhibition restores IKAROS’ ability to regulate *MTOR* expression in primary high-risk B-ALL with deletion of one IKZF1 allele. **a** *MTOR* mRNA level was measured by qRT-PCR in primary high-risk B-ALL samples following treatment with 10 μM CK2 inhibitor (CX-4945) for 2 days as compared to untreated (CTL) cells. **b**–**d** qChIP analysis of (**b**) IKAROS (**c**) H3K27me^3^, and (**d**) H3K9ac at the *MTOR* promoter. Untreated cells are (white and light gray bars) compared to and CX-4945-treated primary high-risk B-ALL (dark gray and black bars). Patients 1, 5, and 6 are Hispanic/Latino. Graphed data are the mean ± SD of combined values from three independent experiments. *p < 0.05, ***p < 0.001, ****p < 0.0001.

Hispanic/Latino children have increased incidence of B-ALL with deletion of one *IKZF1* allele, along with an upregulation of the PI3K/AKT/mTOR pathway and a Ph-like gene expression profile [24, 41]. We tested whether *IKZF1* haploinsufficiency affects IKAROS’s ability to regulate *MTOR* expression and whether CK2 inhibition can regulate the expression of *MTOR* in primary B-ALL cells with deletion of one *IKZF1* allele, including three samples from Hispanic/Latino patients (Table S1). CK2 inhibition with CX-4945 resulted in transcriptional repression of the *MTOR* gene in B-ALL cells from all five B-ALL patients (Fig. 4a). In the B-ALL patient samples that lack one *IKZF1* allele, the IKAROS protein does not bind the *MTOR* promoter (Fig. 4b, light gray bars vs. white bars). CK2 inhibition with CX-4945, restores IKAROS binding in these cells (Fig. 4b, black vs. dark gray bars), and results in the formation of repressive chromatin, characterized by H3K27me^3^ enrichment (Fig. 4c), and loss of H3K9ac (Fig. 4d) at the *MTOR* promoter.

Together, these data demonstrate that CK2 inhibition in high-risk B-ALL cells with the deletion of one *IKZF1* allele, including those from Hispanic/Latino children, restores IKAROS’ ability to bind DNA, induces formation of repressive chromatin at the *MTOR* promoter, and represses transcription of *MTOR*.

### IKAROS and CK2 regulate sensitivity of B-ALL to rapamycin

**Fig. 5:**
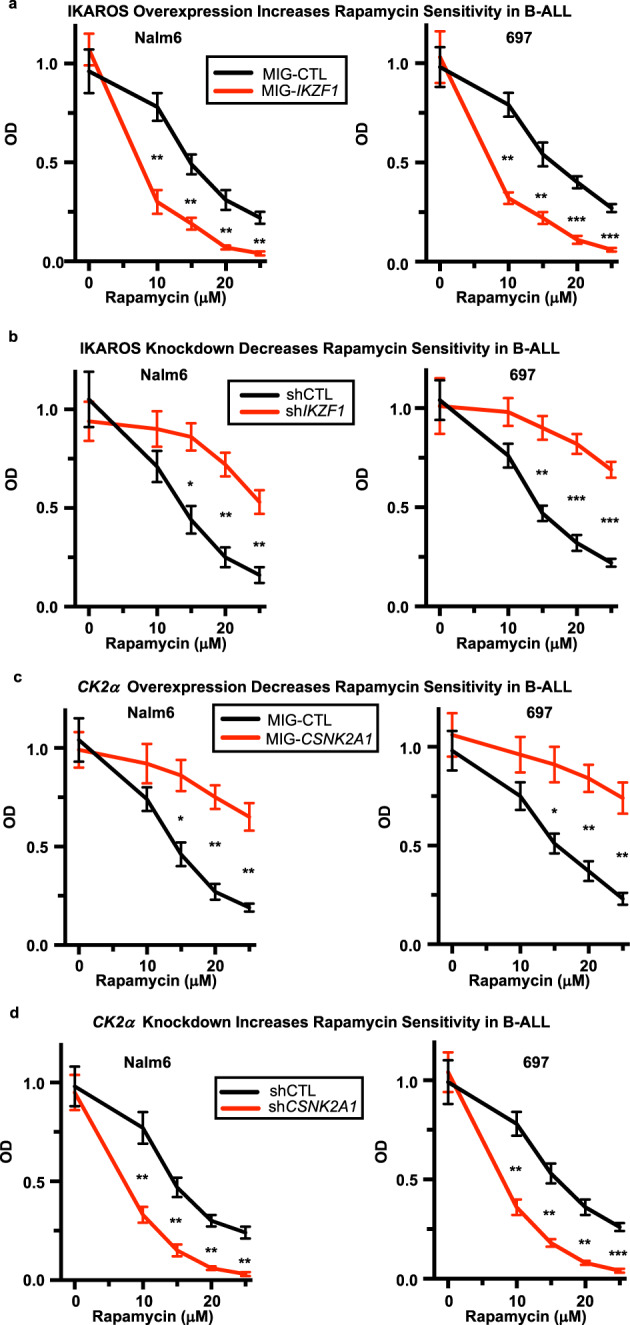
IKAROS and CK2 regulate sensitivity to rapamycin in B-ALL cells. **a** B-ALL cells, retrovirally transduced with *IKZF1 (*MIG*-IKZF1)* or a control vector (MIG-CTL), were FACS-sorted and treated for 3 days with indicated doses of rapamycin and assayed using the WST-1 cell proliferation assay. **b** B-ALL cells, transduced with lentiviral *IKZF1* shRNA (sh*IKZF1*) or scramble shRNA control (shCTL), were FACS-sorted and treated for 3 days with indicated doses of rapamycin. **c**–**d** B-ALL cells with retroviral: (**c**) CK2α overexpression (MIG-*CSNK2A1*) or vector only control (MIG-CTL); or (**d**) lentiviral CK2α shRNA (sh*CSNK2A1*) or scramble shRNA control (shCTL), were FACS-sorted and treated with indicated doses of rapamycin for 3 days then evaluated by WST-1 proliferation assay. Graphed data are the mean ± SD of combined values from three independent experiments. *p < 0.05, **p < 0.01, ***p < 0.001.

Increased mTOR expression and/or activation are associated with poor outcomes in ALL [9, 10]. Therapeutic effects of the mTOR inhibitor, rapamycin, have been tested in clinical trials [8, 42–45]. Since our data demonstrated that IKAROS represses expression of the *MTOR* gene, we tested the effect of IKAROS expression on sensitivity to rapamycin treatment in B-ALL. *IKZF1* overexpression resulted in increased sensitivity to rapamycin (Fig. 5a). Knockdown of *IKZF1* with shRNA resulted in reduced sensitivity to rapamycin (Fig. 5b). These results show that IKAROS expression directly correlates with sensitivity to treatment with rapamycin.

Because CK2 inhibition reduces *MTOR* transcription via IKAROS, we tested the effect of CK2 on the sensitivity of B-ALL cells to rapamycin treatment. Overexpression of CK2α via retroviral transduction resulted in reduced sensitivity to rapamycin-induced cytotoxicity in B-ALL cells (Fig. 5c). Correspondingly, knockdown of CK2α with shRNA resulted in an increased cytotoxic effect of rapamycin treatment in B-ALL (Fig. 5d).

Overall, the presented data demonstrate that the cytotoxic effects of rapamycin are regulated by CK2 and IKAROS expression. The data also suggest that reduced CK2 activity and/or increased IKAROS function increases the therapeutic effect of rapamycin on B-ALL cells.

### The CK2 inhibitor, CX-4945, synergizes with rapamycin in the treatment of B-ALL cells

**Fig. 6:**
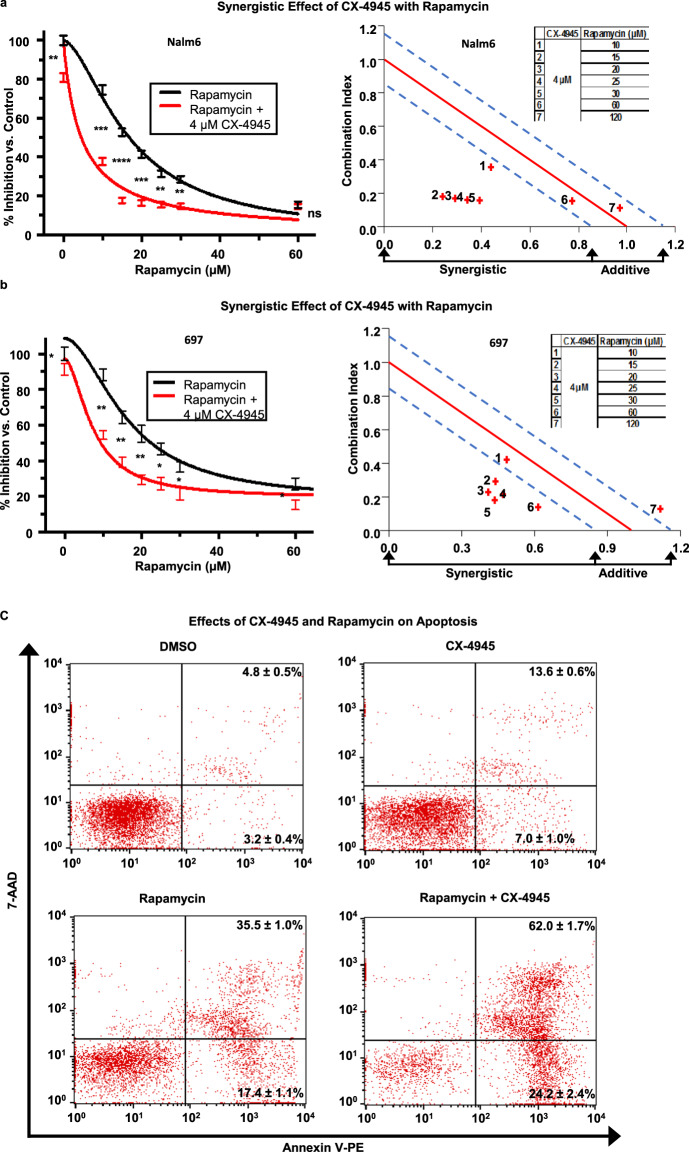
Synergistic effects of CX-4945 with rapamycin on cellular proliferation and apoptosis in B-ALL cells. **a**–**b** Effects (left panels) and synergistic analysis (right panels) of rapamycin (black line) and the combination of rapamycin and CX-4945 (red line) on proliferation of (**a**) Nalm6 cells and (**b**) 697 cells. Cells were treated with the indicated drugs for 2 days. Cellular proliferation was measured by WST-1 assay. Synergistic analysis was performed using Calcusyn, *Y* axis is the combination index (CI) value. CI value is: 0.85–1.15, additive effect, 0.7–0.85, moderately synergistic; <0.7, very synergistic effect (**c**) Effect of CX-4945 (4 μM), rapamycin (10–20 μM), and the combination of CX-4945 (4 μM) plus rapamycin (10–20 μM) on apoptosis in Nalm6 B-ALL cells. Cells were treated for 2 days and stained with 7-AAD and annexin V for flow cytometry to assess apoptosis. The percentage of cells in the lower right quadrant and upper right quadrant of each flow chart represents the percentage of early apoptotic or late apoptotic cells, respectively, in samples treated with the indicated drugs. *p < 0.05, **p < 0.01, ***p < 0.001, ****p < 0.0001.

Because the data presented in Fig. 5 demonstrate that inhibition of CK2 increases the cytotoxic effects of rapamycin on B-ALL, we tested whether the combination of CK2 inhibitor and rapamycin exert synergistic therapeutic effects on B-ALL cells. We used the CK2 inhibitor, CX-4945 [46], that is currently being tested in a Phase I trial [47], and compared the therapeutic effect of CX-4945 and rapamycin combination therapy vs. single-drug treatment, in vitro, on two different human B-ALL cell lines, Nalm6 and 697. Drug response and synergy analyses show that the combination of CX-4945 and rapamycin, given at doses that are achievable in patient serum, produced synergistic cytotoxic effects in both Nalm6 (Fig. 6a) and 697 (Fig. 6b) cell lines.

Combination treatment with CX-4945 and rapamycin, induced increased apoptosis of Nalm6 B-ALL cells as compared to treatment with either drug alone (Fig. 6c).

Overall, these data demonstrate that combination treatment with CX-4945 and rapamycin exerts synergistic cytotoxic activity in B-ALL, and that one mechanism responsible for this synergistic effect involves augmenting the pro-apoptotic effect of rapamycin via CK2 inhibition.

### CK2 inhibition augments cytotoxicity of rapamycin in B-ALL patient-derived xenografts from Hispanic/Latino children with high-risk B-ALL

A frequent feature of high-risk B-ALL in Hispanic/Latino children is increased activity of the PI3K/AKT/mTOR pathway, which is associated with resistance to chemotherapy and poor prognosis [33, 40]. We hypothesized that targeting the PI3K/AKT/mTOR pathway in this type of B-ALL would exert a strong therapeutic effect in vivo. The in vitro synergistic cytotoxic effects of combination treatment with the CK2 inhibitor, CX-4945, and rapamycin, in B-ALL, suggest that CX-4945 could augment the therapeutic activity of rapamycin against B-ALL in vivo. We tested the therapeutic effect of combination treatment with CX-4945 and rapamycin vs. single-drug therapy in preclinical models of high-risk B-ALL in Hispanic/Latino children. B-ALL was determined to be high-risk based on negative prognostic markers (e.g., deletion of one *IKZF1* allele, CRLF2 overexpression etc.) and/or clinical features (Table S1). Following engraftment, mice from each PDX were divided into the following four treatment groups: Group 1: vehicle control, Group 2: CX-4945, Group 3: rapamycin, and Group 4: CX-4945 plus rapamycin combination treatment. Following the completion of treatment, the total live leukemia cells in BM and spleen of mice was determined by flow cytometry.

**Fig. 7:**
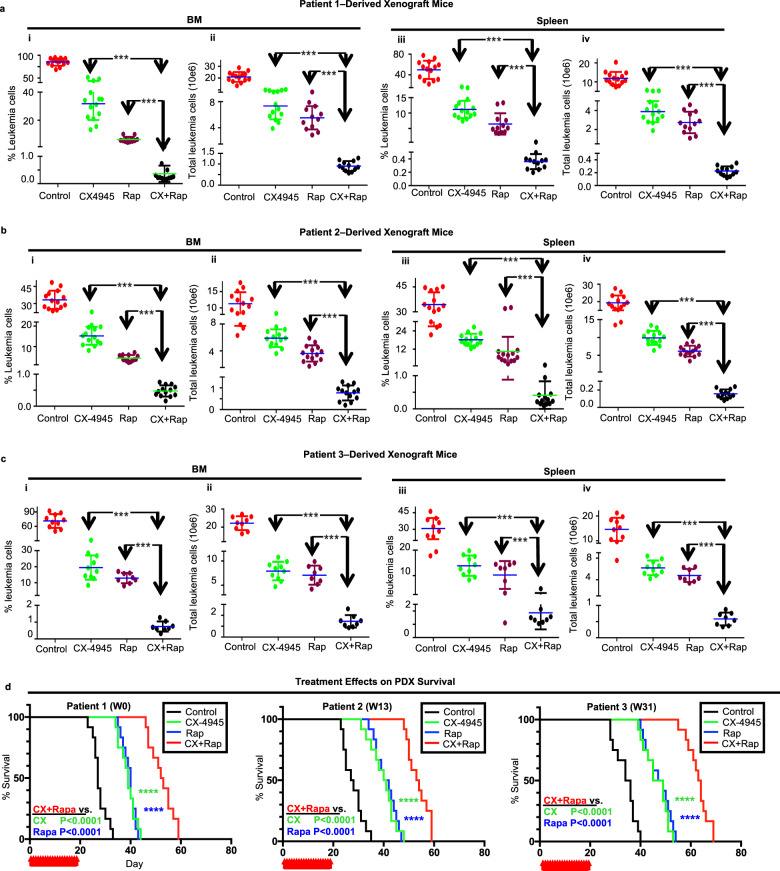
CK2 inhibition augments cytotoxicity of rapamycin in patient-derived xenografts (PDX) from Hispanic/Latino children with high-risk B-ALL. NRG mice were transplanted *via* tail vein with primary B-ALL cells from three Hispanic/Latino patients. Once engraftment was established mice were treated with CX-4945 only, rapamycin (Rap) only, CX-4945 + Rap or with vehicle-only control. **a**–**c** Following euthanasia, BM and spleen cells were counted, and stained for flow cytometry to detect human B cell markers (CD10 and CD19), mouse CD45, and 7-AAD as a dead cell marker. The percentage of the living B-ALL leukemia cells (i, iii) and total leukemia cells (ii, iv) in BM and spleen were calculated and graphed. The effect of drug treatment was assessed by student’s *t* test. **d** Patient-derived xenografts established with B-ALL from Patients 1–3 were treated for 24 days with CX-4945, rapamycin (Rap) only, CX-4945 plus rapamycin (CX + rap) or vehicle control and followed for survival. Survival curves were generated using the Kaplan–Meier method and differences in survival were analyzed by Chi-square test. ***p < 0.001, ****p < 0.0001.

Results showed that combination treatment with CX-4945 and rapamycin produced a significantly stronger therapeutic effect in all three PDX models, compared to single-drug therapy and/or control (Fig. 7a–c and Fig. S10–12). The total number of viable leukemia cells was severely reduced (3–4-fold) in the BM and spleen of the PDX mice treated with combination therapy, compared to mice treated with CX-4945 or rapamycin alone. These results demonstrate that the combination of CK2 kinase inhibitor (CX-4945) with rapamycin has a synergistic therapeutic and cytotoxic effect on high-risk B-ALL cells from Hispanic/Latino children, when given as a combination treatment, in vivo.

Laboratory analysis showed that complete blood count, and kidney function tests were not affected by treatment with CX-4945 or rapamycin alone, or in combination (data not shown).

The effect of CX-4945 treatment on *MTOR* transcription during in vivo treatment was analyzed before cytotoxicity occurred (at days 3 and 7 following the initiation of in vivo treatment with single drugs or combination therapy with CX-4945 and rapamycin, as described above). Data showed that in vivo treatment of primary xenografts with the CK2 inhibitor, CX-4945, in combination with rapamycin, results in the inhibition of mTOR pathway and reduced transcription of the *MTOR* gene in leukemia cells in both BM and spleen (Fig. S13).

Mice were followed for survival using the Kaplan–Meier method. Results showed that combination treatment with CX-4945 and rapamycin significantly prolongs survival of mice, compared to single-drug treatment (Fig. 7d).

Together, these results demonstrate that combination treatment with the CK2 inhibitor, CX-4945, and rapamycin has a superior therapeutic effect in preclinical models of high-risk B-ALL of Hispanic/Latino children, compared to single-drug treatment. These results suggest that one of the mechanisms through which CK2 inhibition augments the therapeutic effect of rapamycin in vivo includes transcriptional repression of *MTOR*.

## Discussion

The presented data show that IKAROS functions as a transcriptional repressor of *MTOR*. Phosphorylation by the oncogenic kinase, CK2, abolishes IKAROS’ function as a transcriptional regulator [37, 48–56]. In high-risk B-ALL, the function of IKAROS as an *MTOR* repressor is impaired by the deletion of one *IKZF1* allele, or by IKAROS inactivation due to phosphorylation by CK2, which is overexpressed in B-ALL [5, 37]. Inhibition of CK2 restores IKAROS binding to the *MTOR* promoter and transcriptional repression of *MTOR*. Previously published data showed that IKAROS represses the transcription of genes that are essential for the PI3K pathway and that CK2 directly inactivates PTEN through phosphorylation [5, 37, 57]. Results presented in this report reveal a novel mechanism through which CK2 and IKAROS regulate PI3K/AKT/mTOR signaling in B-ALL—via regulation of *MTOR* transcription.

mTOR is a kinase that promotes cellular proliferation and survival [58]. Upregulation of the PI3K/AKT/mTOR pathway results in the development of high-risk leukemia that is resistant to chemotherapy [6, 10, 59]. Children of Hispanic/Latino ancestry have an increased frequency of high-risk B-ALL and worse overall survival when compared to other racial/ethnic groups [1]. Molecular epidemiological studies demonstrated increased incidence of translocations resulting in the overexpression of the *CRLF2* gene in Hispanic/Latino pediatric patients [2, 33]. Our group reported that the deletion of an *IKZF1* allele, alone, or in combination with the *CRLF2* translocation, is significantly increased in B-ALL of Hispanic/Latino children^COMPANION PAPER^. CRLF2 acts as an upstream activator of the PI3K/AKT/mTOR pathway [4]. Thus, the presence of *CRLF2* translocation and/or *IKZF1* deletion makes the increased activity of the PI3K/AKT/mTOR pathway a prominent feature of B-ALL in Hispanic/Latino children, and mTOR an attractive target for treatment of this disease. Our data show that CK2 inhibition with CX-4945 restores IKAROS binding to the *MTOR* promoter, as well as transcriptional repression of *MTOR* by IKAROS in high-risk B-ALL cases where there is a deletion of one *IKZF1* allele. These data provide a rationale for a novel, rationally-designed, mechanism-based, dual approach to target mTOR in high-risk B-ALL in Hispanic/Latino children. This includes the inhibition of mTOR protein function with rapamycin, in combination with the CK2 inhibitor, CX-4945, which represses *MTOR* transcription by enhancing IKAROS repressor activity. The dual targeting of mTOR showed a synergistic effect in vitro, and in vivo using three different preclinical models of high-risk B-ALL generated from Hispanic/Latino children (Figs. 6 and 7).

**Fig. 8:**
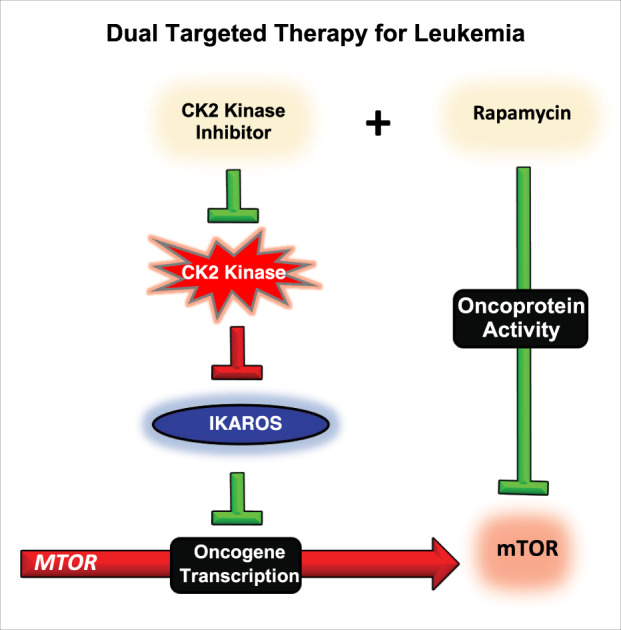
Model of dual targeted therapy for hematological malignancies. Red arrows and bars indicate pro-oncogenic pathways. Green bars indicated anti-oncogenic pathways.

These studies establish a new paradigm for dual-targeting of an oncogenic signaling pathway—targeting both the oncoprotein activity with a direct inhibitor, and the transcription of the gene encoding the oncoprotein (Fig. 8). This dual-targeting approach should overcome chemoresistance due to oncogene overexpression and/or mutation, which often occurs following targeted inhibition. Since the transcriptional regulatory networks of many oncogenes are well-established, this approach opens new possibilities for targeted combination therapies.

In conclusion, our presented data establish the therapeutic efficacy of a novel combination treatment that targets the PI3K/AKT/mTOR signaling pathway in high-risk B-ALL in Hispanic/Latino children. The approach proposed in the study—targeting the transcriptional regulatory network of an oncogene, combined with specific inhibition of the corresponding oncoprotein—can provide a paradigm for similar targeted combination treatments for other hematological malignancies.

## Supplementary information

Supplemental material
