# Supplementary material for: Dual targeting of MTOR as a novel therapeutic approach for high-risk B-cell acute lymphoblastic leukemia
Source: Leukemia. 2021 Feb 2;35(5):1267–78. doi: 10.1038/s41375-021-01132-5 (PMC8102195; doi:10.1038/s41375-021-01132-5)
Supplement: Supplementary file 1 — Supplemental material [file 41375_2021_1132_MOESM1_ESM.docx]

# Supplemental Materials

**Dual Targeting of MTOR as a Novel Therapeutic Approach for High-Risk B-cell Acute Lymphoblastic Leukemia**

Zheng Ge*^1,2^, Chunhua Song***^1,3^**, Yali Ding*^1^, Bi-Hua Tan^1^, Dhimant Desai^1^, Arati Sharma^1^, Raghavendra Gowda^1^, Feng Yue^1^, Suming Huang^1^, Vladimir Spiegelman^1^, Jonathon L. Payne^4,1^, Mark E. Reeves^4^, Soumya Iyer^1^, Pavan Kumar Dhanyamraju^1^, Yuka Imamura^1^, Daniel Bogush^1^, Yevgeniya Bamme^1^, Yiping Yang**^3^**, Mario Soliman^1^, Shriya Kane^1^, Elanora Dovat^1^, Joseph Schramm^1^, Tommy Hu^1^, Mary McGrath^1^, Zissis C. Chroneos^1^, Kimberly J. Payne^4^, Chandrika Gowda^1,#^ and Sinisa Dovat^1,#^

Author Affiliation:

1 Pennsylvania State University College of Medicine, Hershey, PA 17033 ^2^Zhongda Hospital, Medical School of Southeast University Nanjing 210009, China 3Ohio State University College of Medicine, Columbus OH, 43210

4Loma Linda University College of Medicine, Loma Linda CA, 92350

* These authors contributed equally to this work

#To whom correspondence should be addressed:

Sinisa Dovat, MD, PhD, Division of Pediatric Hematology/Oncology, Department of Pediatrics, Pennsylvania State University College of Medicine, Hershey, PA17033.

E-mail: [sdovat@pennstatehealth.psu.edu](mailto:sdovat@pennstatehealth.psu.edu)

Chandrika Gowda, M.D., Division of Pediatric Hematology/Oncology, Department of Pediatrics, Pennsylvania State University College of Medicine, Hershey, PA17033.

E-mail: [cgowda2@pennstatehealth.psu.edu](mailto:cgowda2@pennstatehealth.psu.edu)

#### Approval for animal studies and use of patient samples

Supplemental Methods

All animal experiments were conducted in the Developmental Therapeutics Preclinical Core facility at Penn State University College of Medicine under protocols approved by the Institutional Animal Care and Use Committee at Penn State Hershey, Hershey, PA. Deidentified patient samples were provided by Loma Linda University (Loma Linda, CA) and the University of Southern California Norris Comprehensive Cancer Center (Los Angeles, CA) and used in compliance with Institutional Review Board regulations. A summary of patients’ characteristics is in Table S1.

#### Cell culture and reagents

CX-4945 was purchased from AdooQ Bioscience (CA, USA). Rapamycin, and 4,5,6,7- Tetrabromo-2-azabenzimidazole, 4,5,6,7-Tetrabromobenzotriazole (TBB) were purchased from Millipore-Sigma **(**St. Louis, USA).

The Nalm6 B-ALL cell line and 293T cells have been described previously.^1-5^ The 697 (EU-3) are from DSMZ (Braunschweig, Germany).^1,^ ^2^ All of the cells are tested for mycoplasma contamination every 3 months.

Nalm6 and 697 cells were grown in RPMI 1640 (Mediatech, Manassas VA) with 10% heat- inactivated Fetal bovine serum (FBS) (HyClone, Rockford, IL), 1% penicillin-streptomycin and 1% L- glutamine until exponential growth was obtained. N6/ADR cells were cultured in the same media as Nalm6 cells in the presence of 70nM of Doxorubicin. For CX-4945 or TBB treatment, cells were cultured for 2 days.

Primary human B-ALL cells were co-cultured with or without a mixture of human HS-27 stroma (American Type Culture Collection [ATCC]; Rockville, MD) and murine MS-5 stromal cells as described previously.^1,^ ^2,^ ^6^ Cells were cultured with or without CX-4945 or TBB and collected for total RNA isolation, and for ChIP-qPCR assay. Wild type *IKZF1* or *IKZF1* haploinsufficiency in patient samples was confirmed by Western blot and/or DNA sequencing.

#### ChIP-Seq experiments

ChIP-Seq assays for IKAROS in Nalm6, 697, JM-1, REH B-ALL cells and patient 9 sample, and also JM-1 cells (20uM drugs for 1 day) were performed as previously reported.^1-3,^ ^7-9^ Briefly, the ChIP assay was performed with affinity purified anti-IKAROS-CTS antibody^1,^ ^10^ (A303-516A, Bethyl) by incubation with chromatin prepared from the cells. Libraries were prepared and sequenced on an Illumina NovaSeq 6000 (Illumina). The sequencing reads were de-multiplexed, adapter-trimmed, filtered, and aligned to the human reference genome (GRCh38). Peaks were called using MACS2 (version 2.1.1) and homer (version 4.10) by supplying GRCh38.78.gtf as the annotation file. Custom tracks for UCSC Browser were generated by converting the peak pileup bedGraph files generated by the MACS2 to bigwig files and setting the viewLimits to 40 for the pileup heights. The custom tracks for UCSC Browser were shown by converting the peak pileup bedGraph files precessed with the same approaches.

IKAROS ChIP-seq signal plots for Primary B-ALL from Patient 9 and from Nalm6 cells used previously published data (see link below) and the custom tracks for CisGenome Browser were generated with reference genome (GRCh37).

Primary B-ALL ChIP-Seq data from Patient 9 are available on Gene Expression Omnibus with an access number of GSE58825 at the following link: https://[www.ncbi.nlm.nih.gov/geo/query/acc.cgi?acc=GSE58825](http://www.ncbi.nlm.nih.gov/geo/query/acc.cgi?acc=GSE58825)

Nalm6 ChIP-Seq data are accessible on Gene Expression Omnibus with an access number of GSE44218 at the following link: [http://www.ncbi.nlm.nih.gov/geo/query/acc.cgi?acc=GSE44218.](http://www.ncbi.nlm.nih.gov/geo/query/acc.cgi?acc=GSE44218)

Remaining ChIP-Seq data are accessible on Gene Expression Omnibus with an accession number of GSE141572 at the following link: https://[www.ncbi.nlm.nih.gov/geo/query/acc.cgi?&acc=GSE141572](http://www.ncbi.nlm.nih.gov/geo/query/acc.cgi?&acc=GSE141572) (Reviewer security token: ydspaokitpaldsr).

#### Plasmid construction and retroviral gene transfer

Wild-type human HA-tagged *IKZF1* cDNA was cloned at BglII and EcoRI sites into the pMSCV bicistronic retroviral vector (MIG vector) which contains a 5’ long-terminal-repeat-driven *IKZF1*, internal ribosome entry site (IRES), and enhanced green fluorescent protein (EGFP). Retroviruses were produced by transient transfection in amphotropic packaging HEK293T cell lines as described previously.^1-3^ Retrovirus was concentrated using the Retro-X^TM^ concentrator (Clontech Laboratories, Inc.) according to the manufacturer's instructions. Nalm6 or 697 cells were plated in 24-well plates at 4x10^5^ cells/well and suspended in retroviral supernatants with 12.5 μg/ml polybrene and centrifuged 1,400 *g*, at 32 °C, for 2h as previously described.^1-3^ Cells were then suspended in fresh 10% FBS RPMI 1640 and cultured at 37°C, in a 5% CO2 incubator for 3 days. The cells were Ficolled and the GFP(+) cells were sorted with a FACSAria High speed cell sorter (Becton Dickinson). The sorted cells were cultured for further biochemistry assays.

***IKZF1* and *CK2α* shRNA knockdown**

*IKZF1* knockdown was performed with lentiviral shRNA expression as described previously.^1-3,^ ^9,^ 11, 12 Briefly, Nalm6 and 697 cells were infected with the lentivirus and sorted seventy-two hours after infection. The sorted cells were cultured for further biochemistry assays as detailed below. *CK2α* shRNA knockdown was performed using the Neon Transfection System (Invitrogen) as previous reported.^1-3,^ ^9,^ ^11,^ ^12^ *IKZF1* shRNA knockdown was also performed using this method. Briefly, 29mer shRNA constructs for human *IKZF1* and human *CK2α* (*CSNK2A1*) in a GFP vector (pGFP- V-RS) were purchased from Origene (Rockville, MD, USA). Nalm6 or 697 cells were transiently transfected with the shRNA plasmids or scramble shRNA control using the transfection system. After transfection for 1 day, Nalm6 or 697 cells with transfection efficiency ranges from ~80% (green cells) and more than 95% cell viability were further treated with 20μM CX-4945 or vehicle control (0.01% DMSO) for 2 days and harvested for total RNA isolation and total lysate extraction. The 29-mer scrambled shRNA cassette in pGFP-V-RS vector was also used as a control. Knockdown of *IKZF1* and *CK2* was confirmed by qRT-PCR measurement of *IKZF1* mRNA levels and Western blot measurement of IKAROS protein levels using anti-IKAROS-CTS antibody as reported previously.^1-^

3, 13, 14

#### Quantitative ChIP-qPCR (qChIP) assay

IKAROS qChIP assays ware performed as described previously^1-3,^ ^9,^ ^10,^ ^12^ by incubation of the chromatin with anti-IKAROS antibody or normal rabbit IgG (Abcam, ab46540) as a control. Enrichment of the ChIP sample over input (% input and fold enrichment) was evaluated by qPCR with three or more replicates, using specific primers in the promoter region of the *MTOR* gene. Primers’ sequences are: forward: 5’-CCGCGTGGTTTGTCTATTTGA-3’, Reverse: 5’- CTTCAGGACCCGGCTT CTC-3’. Histone modification marker qChIP assays were carried out with the same protocol as IKAROS qChIP by incubating chromatin with anti-H3K927me^3^ (Millipore,07- 449) or H3K9ac (Abcam, Ab4441) antibody or anti-H3K4me^3^(Abcam, ab8580) or normal rabbit IgG (Abcam, ab46540) as a control.

#### qRT-PCR assays of gene expression

The mRNA expression of the *MTOR* gene was evaluated by qRT-PCR assay as previously reported.^1-3,^ ^9,^ ^10,^ ^12^ Briefly, total RNA (2.0 μg total RNA was reverse transcribed using poly d(T)20 primers and SuperScript II reverse transcriptase (Invitrogen, Walthan, MT, USA). The resulting cDNAs were used for qPCR analysis of their mRNA levels on a StepOne Plus 7500 Real-time PCR system (Applied Bioscience Inc., Foster City, CA) using specific primers for the *MTOR* gene with the primers’ sequences (forward: 5’- TGCTGAACTGGAGGCTGATG-3’; Reverse: 5’- TCGTTCGGGATCGCTTGT-3’) following the manufacturer's instructions. The fluorescence threshold value was calculated and normalized to the values of 18s rRNA. The fold change in mRNA expression of genes between the treatment (drug, IKAROS expression and shRNA) group versus control group (none treatment, vector only or scramble shRNA) was achieved by the ratio of fluorescence threshold value in treatment or nontreatment group. The fold change in gene expression are depicted in bar graphs.

#### Luciferase assays of promoter activity

The LightSwitch transfection-ready luciferase reporter construct for the *MTOR* promoter was purchased from SwitchGear Genomics. The transient luciferase assay was performed as previously described.^1,^ ^2,^ ^9^ Luciferase activities were calculated as fold change relative to values obtained from pLightSwitch-Rom vector only control cells, and expressed as a percentage of pcDNA3.1-IKAROS transfection-induced luciferase activity versus that of pcDNA3.1 vector. All transfection and reporter assays were performed independently, in triplicate, at least three times.

#### Proliferation and cytotoxicity assays

The colorimetric WST-1 cell proliferation assay (Roche Applied Science; 11644807001) was performed in 96-well white clear bottom plates (Costar, 3610) in quadruplicate, according to manufacturer’s instructions. Absorbance at 440 nm (reflects number of viable cells) was measured using a BioTek Synergy Mx plate reader. Toxicity assays were performed in triplicate by incubating 1.0×10^6^ cells per well in a 24-well plate in the presence or absence of drugs in a final volume of 1 ml and used to calculate synergistic drug interactions. Aliquots of cells were harvested at indicated time periods and hemocytometer count using Trypan blue exclusion was used to obtain viable cell counts.

#### Apoptosis assays

Apoptosis assays were performed as described previously.^1,^ ^2,^ ^9^ Cells were stained using an Apoptosis Detection Kit (Affymetrix-eBioscience) following the manufacturer’s instructions. Briefly, 3 × 10^5^ Nalm6 cells were treated with CX-4945 for 48 h. The cells were then harvested, washed in PBS and stained with Annexin V PE and 7-AAD in binding buffer at room temperature for 10 min in the dark. The stained cells were analyzed using a FACSCalibur instrument.

#### Antibodies, cell lysates and Western blot

Lysates from cells treated with the CK2 inhibitor, CX-4945, or vehicle control were prepared on ice in RIPA lysis buffer (50 mM Tris HCl pH 7.4, 150 mM NaCl, 0.1% SDS and 1% NP40) including a protease inhibitor cocktail.^1,^ ^2,^ ^9^ Protein was quantified using the Bradford assay and used for Western blot analysis and immunoblot. Western blots were performed using antibody: anti-mTOR, p-AKT Thr308, Cat # 9275; p-4EBP1, cat # 2855; phospho p70S6K Cat # 9208(Cell Signaling Technology, Danvers, MA, USA) or anti-Actin (Sigma, St. Louis, MO, USA). Antibodies for qChIP were: anti-H3K927me3 (Millipore, 07- 449) or H3K9ac (Abcam, Ab4441) antibody or anti-H3K4me3 (Abcam, ab8580) or normal rabbit IgG (Abcam, ab46540) (Cell Signaling Technology, Danvers, MA, USA) or anti-Actin (Sigma, St. Louis, MO, USA).

#### High-risk B-ALL patient-derived xenograft models

All animal experiments were conducted in the Developmental Therapeutics Preclinical Core facility at Penn State University College of Medicine under protocols approved by the Institutional Animal Care and Use Committee at Penn State Hershey, Hershey, PA. Deidentified patient samples were provided by Loma Linda University (Loma Linda, CA) and the University of Southern California Norris Comprehensive Cancer Center (Los Angeles, CA) and used in compliance with Institutional Review Board regulations. For the primary human B-ALL mouse xenograft model, 2x10^6^ patient B-ALL cells per mouse were transplanted intravenously into 4-week-old female NOD.Cg-*Rag1^tm1Mom^ Il2rg^tm1Wjl^*/SzJ (NRG) mice. The sample size was n = 14/group/per patient sample x 3 patients. The sample size was chosen based on Power analysis for a one-way ANOVA, which showed that animal numbers will be sensitive enough to detect a minimum effect size of .46 with α = 0.05, β = 0.2, F= 3.20; calculated using G*Power 3.1.9.2 Following engraftment, animals were randomly selected from the pool of engrafted animals and assigned to the control or experimental groups. Mice received: vehicle (Group1), CX-4945 daily *via* gavage at 100 mg/kg/day (Group 2); rapamycin 4 mg/kg IP once a week (Group 3) and combination treatment with CX-4945 and rapamycin at the same doses as single drug groups, given simultaneously (Group 4) until death of the first animal in experiment (3-5 weeks). The animal technician administering drugs to mice was blinded to the group allocation. Determination of engraftment for treatment initiation was based on the presence of >25% human leukemia cells in total BM mononuclear cells as reported previously.^2^ Following treatment period, a single cell suspension was prepared from harvested BM and spleen from euthanized mice, and red blood cells (RBCs) were lysed using RBC lysis buffer (Biolegend). Resulting cells were used for living cell counts, quantitative ChIP-qPCR (qChIP) assay, quantitative reverse transcriptase PCR (qRT-PCR), and flow cytometry analysis. For survival studies, animals were treated for 24 days.

#### Flow cytometry for engraftment analysis of human leukemia

Aliquots of single cell suspensions from BM or spleen of each mouse were blocked with human CD16/32 antibodies (Biolegend) and then stained for flow cytometry with antibodies specific to mouse CD45-FITC (mCD45), human CD19-APC and human CD10-PE (Biolegend). The resulting cells were analyzed by flow cytometry (BD LSR Fortessa). Live cells were discriminated by staining with 7-AAD (Biolegend). Leukemia cell engraftment was the percentage of cells that were mCD45– and double positive for CD19+ and CD10+ (human B cells) within the total living cell (7-AAD– and living cell light scatter) gate. Leukemia cell numbers were obtained by multiplying the leukemia cell engraftment percentage obtained for the BM or spleen by the total living cell count in respective tissues.

#### Statistical analyses

Data were represented as mean value with bars representing the standard deviation (SD). Determinations of statistical significance were performed using an unpaired two-tailed Student *t*-test for comparisons of two groups or using analysis of variance (ANOVA) for comparing multiple groups. Data were analyzed and plotted with GraphPad Prism 3.03 software (GraphPad Software, Inc., La Jolla, CA, USA).

For combination index (CI) determination, the effect of non-constant ratios of combinations of CX-4945 and rapamycin, CalcuSyn software version 1.0 (Biosoft, Ferguson, MO, USA) was used. The CI plot was then obtained, according to the Chou-Talalay method.^15^

#### References

1. Song C, Ge Z, Ding Y, Tan BH, Desai D, Gowda K*, et al.* IKAROS and CK2 regulate expression of BCL-XL and chemosensitivity inhigh-risk B-cell acute lymphoblastic leukemia. *Blood* 2020 May 12.
2. Song C, Gowda C, Pan X, Ding Y, Tong Y, Tan BH*, et al.* Targeting casein kinase II restores Ikaros tumor suppressor activity and demonstrates therapeutic efficacy in high-risk leukemia. *Blood* 2015 Oct 8; **126**(15)**:** 1813-1822.
3. Song C, Pan X, Ge Z, Gowda C, Ding Y, Li H*, et al.* Epigenetic regulation of gene expression by Ikaros, HDAC1 and Casein Kinase II in leukemia. *Leukemia* 2016 Jun; **30**(6)**:** 1436-1440.
4. Findley HW, Jr., Cooper MD, Kim TH, Alvarado C, Ragab AH. Two new acute lymphoblastic leukemia cell lines with early B-cell phenotypes. *Blood* 1982 Dec; **60**(6)**:** 1305-1309.
5. Campana D, Janossy G, Bofill M, Trejdosiewicz LK, Ma D, Hoffbrand AV*, et al.* Human B cell development. I. Phenotypic differences of B lymphocytes in the bone marrow and peripheral lymphoid tissue. *J Immunol* 1985 Mar; **134**(3)**:** 1524-1530.
6. Parrish YK, Baez I, Milford TA, Benitez A, Galloway N, Rogerio JW*, et al.* IL-7 Dependence in human B lymphopoiesis increases during progression of ontogeny from cord blood to bone marrow. *J Immunol* 2009 Apr 1; **182**(7)**:** 4255-4266.Wang Z, Zang C, Rosenfeld JA, Schones DE, Barski A, Cuddapah S*, et al.* Combinatorial patterns of histone acetylations and methylations in the human genome. *Nat Genet* 2008 Jul; **40**(7)**:** 897-903.
7. Fujiwara T, O'Geen H, Keles S, Blahnik K, Linnemann AK, Kang YA*, et al.* Discovering hematopoietic mechanisms through genome-wide analysis of GATA factor chromatin occupancy. *Mol Cell* 2009 Nov 25; **36**(4)**:** 667-681.
8. Wang H, Song C, Ding Y, Pan X, Ge Z, Tan BH*, et al.* Transcriptional Regulation of JARID1B/KDM5B Histone Demethylase by Ikaros, Histone Deacetylase 1 (HDAC1), and Casein Kinase 2 (CK2) in B-cell Acute Lymphoblastic Leukemia. *J Biol Chem* 2016 Feb 19; **291**(8)**:** 4004-4018.
9. Ding Y, Zhang B, Payne JL, Song C, Ge Z, Gowda C*, et al.* Ikaros tumor suppressor function includes induction of active enhancers and super-enhancers along with pioneering activity. *Leukemia* 2019 Nov; **33**(11)**:** 2720-2731.
10. Gowda C, Song C, Kapadia M, Payne JL, Hu T, Ding Y*, et al.* Regulation of cellular proliferation in acute lymphoblastic leukemia by Casein Kinase II (CK2) and Ikaros. *Adv Biol Regul* 2017 Jan; **63:** 71-80.
11. Payne JL, Song C, Ding Y, Dhanyamraju PK, Bamme Y, Schramm JW*, et al.* Regulation of Small GTPase Rab20 by Ikaros in B-Cell Acute Lymphoblastic Leukemia. *Int J Mol Sci* 2020 Mar 3; **21**(5).
12. Gurel Z, Ronni T, Ho S, Kuchar J, Payne KJ, Turk CW*, et al.* Recruitment of ikaros to pericentromeric heterochromatin is regulated by phosphorylation. *J Biol Chem* 2008 Mar 28; **283**(13)**:** 8291-8300.
13. Ott CJ, Federation AJ, Schwartz LS, Kasar S, Klitgaard JL, Lenci R*, et al.* Enhancer Architecture and Essential Core Regulatory Circuitry of Chronic Lymphocytic Leukemia. *Cancer Cell* 2018 Dec 10; **34**(6)**:** 982-995 e987.
14. Pham LV, Tamayo AT, Li C, Bornmann W, Priebe W, Ford RJ. Degrasyn potentiates the antitumor effects of bortezomib in mantle cell lymphoma cells in vitro and in vivo: therapeutic implications. *Mol Cancer Ther* 2010 Jul; **9**(7)**:** 2026-2036.

**Supplemental Tables**

**Supplemental Table 1: Leukemia Patient Characteristics**

| **Patient Designation** | **Gender** | **Age (ped/adult)** | ***IKZF1* Status and Genetic Abnormalities** | **High-Risk B-ALL Features** |
| --- | --- | --- | --- | --- |
| Patient 1 (W0)  Hispanic/Latino | M | 10 years | *IKZF1* deletion | >1x10^6^ leukemia cells/microliter in peripheral blood;  99% blasts; *IKZF1*-deletion; age |
| Patient 2 (W13)  Hispanic/Latino | M | 18 months | No *IKZF1* deletion | 6x10^5^ leukemia cells/microliter in peripheral blood;  95% blasts |
| Patient 3 (W31)  Hispanic/Latino | M | 10 years | No *IKZF1* deletion | CRLF2  overexpression; age |
| Patient 4 (ICN1)  Asian | M | 12 years | No *IKZF1* deletion;  *BCR-ABL* | Age; BCR-ABL |
| Patient 5 (W10)  Hispanic/Latino | M | 18 years | *IKZF1* deletion | >8x10^5^ leukemia cells/microliter in peripheral blood;  97% blasts; *IKZF1*-deletion; 18 years; CRLF2-high |
| Patient 6 (LAX7)  Hispanic/Latino | M | Young adult | *IKZF1* deletion | *IKZF1* deletion; age |
| Patient 7 (MXP3)  Race/Ethnicity Unknown | M | 13 years | *IKZF1* deletion; *PAX5* deletion; *BCR-ABL* | *IKZF1* deletion; BCR- ABL |
| Patient 8  (MXP5)  Race/Ethnicity Unknown | M | 5 years | *IKZF1* deletion;  *PAX5* deletion  exon (2-6); BCR-ABL | *IKZF1* deletion; BCR- ABL |
| Patient 9 (LAX2)  Race/Ethnicity Unknown | M | 38 years | No *IKZF1* deletion;  *BCR-ABL* | Age; *BCR-ABL* |

**Supplemental Figures**

#### a

**60 2 kb**


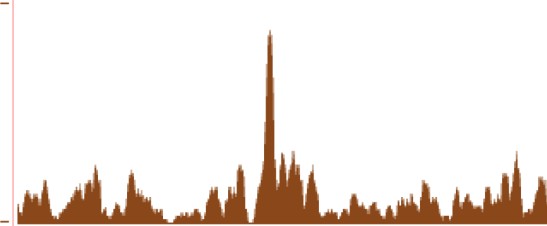

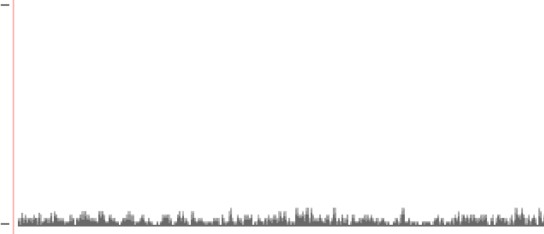


**Input**

**REH**

**b**

**40 2 kb**


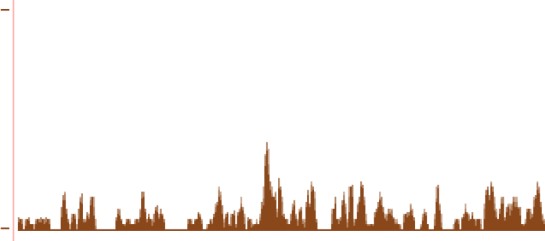

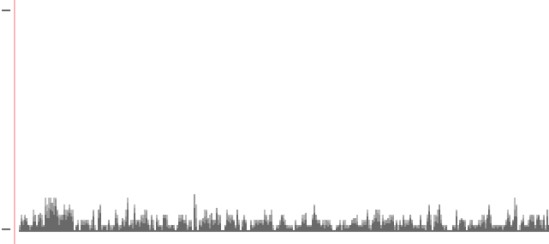


**Input**

## 697

**0 11,260,000 11,265,000 11,270,000**

**60 2 kb**

**IKAROS**

**Chr1**

**0**

**11,260,000 11,265,000**

**40 2 kb**

**IKAROS**

**11,270,000**

**Chr 1**

**0**

**11,260,000**

**11,265,000**

**11,270,000**

**Chr1**

**0**

**11,260,000**

**11,265,000**

**11,270,000**

**Chr 1**

***MTOR***

***MTOR***

**Supplemental Figure S1. IKAROS binds to the *MTOR* promoter**. **(a)** IKAROS binding sites at the *MTOR* promoter identified by ChIP-seq in **(a)** the REH B-ALL cell line; **(b)** the 697 B-ALL cell line. The ChIP-seq data for REH and 697 is analyzed with reference genome MRCh38 and the custom tracks are shown on the UCSC Genome Browser.

**b IKAROS Binds the *MTOR* Promoter in B-ALL Cell Lines 4**

*******

**~~***~~**

*******

***α*-IgG**

***α*-IKAROS**

**ns**

**3**

**Fold enrichment vs. IgG**

**2**

**1**

**0**

**Nalm6 697**

**JM1 293T**

**Supplemental Figure S2. IKAROS binds the *MTOR* promoter**. qChIP analysis shows IKAROS binding at the *mTOR* promoter in human B-ALL cell lines but not HEK293T cells, which have no IKAROS protein expression (negative control). Graphed data are the mean +/– SD of triplicates representative of one of 3 independent experiments. *p<0.05, **p<0.01, ***p<0.001, ****p<0.000 1.

**a *IKZF1* Transcription Levels After IKAROS Overexpression**

**b IKAROS Overexpression Increases IKAROS Binding**

**at the *MTOR* Promoter**

MIG-CTL MIG-*IKZF1*


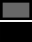


CTL–*α*-IgG CTL–*α*-IKAROS

*IKZF1*–*α*-IgG *IKZF1*–*α*-IKAROS

**8 15**

********

********

*******

********

*******

********

********

********

**Relative *IKZF1* transcription**

**Fold enrichment vs. IgG**

**6**

**10**

**4**

**5**

**2**

**0**

**Nalm6 697**

**c**

***IKZF1* Transcription Levels**

**After IKAROS Knockdown**

shCTL sh*IKZF1*

**0**

**Nalm6 697**

**d IKAROS Knockdown Decreases IKAROS Binding**

**at the *MTOR* Promoter**


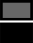


shCTL–*α*-IgG sh*IKZF1*–*α*-IgG shCTL–*α*-IKAROS sh*IKZF1*–*α*-IKAROS

**1.5 4**

********

********

*******

*******

*******

*******

**ns**

**ns**

**Relative *IKZF1* transcription**

**Fold enrichment vs. IgG**

**1.0**

**0.5**

**0.0**

**Nalm6**

**697**

**3**

**2**

**1**

**0 Nalm6**

**697**

**Supplemental Figure S3. IKAROS overexpression or shRNA knockdown affect its binding to the *MTOR* promoter in Nalm6 and 697 B-ALL cells. (a-b)** Cells were retrovirally transduced with IKAROS (MIG-*IKZF1*) or vector control (MIG-CTL). **(a)** Increased transcription of *IKZF1* following retroviral transduction with IKAROS (MIG-*IKZF1*) or vector control (MIG-CTL) was analyzed by qRT-PCR. **(b)** qChIP was performed with indicated antibodies**. (c-d)** Cells were transduced to express IKAROS shRNA (sh*IKZF1)* or scramble shRNA (shCTL) as a control. **(c)** Decreased transcription of IKAROS following transduction with sh*IKZF1* or shCTL was analyzed by qRT-PCR. **(d)** qChIP was performed with indicated antibodies. *p<0.05, **p<0.01, ***p<0.001,

****p<0.0001.

**a**

***CK2α* Expression After**

***CSNK2A1* Knockdown**

**1.5**

*******

*******

shCTL sh*CSNK2A1*

**Relative *CSNK2A1* transcription**

**1.0**

**0.5**

**0.0**

**Nalm6 697**

**b**

**CK2*α* Knockdown Increases IKAROS at *MTOR* Promoter**

**15**

********

********

********

********

********

********

**10**

**Fold enrichment vs. IgG**

**5**


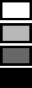


shCTL–*α*-IgG shCTL–*α*-IKAROS sh*CSNK2A1*–*α*-IgG

sh*CSNK2A1*–*α*-IKAROS

**0**

**Nalm6 697**

**Supplemental Figure S4. CK2*α* Molecular Inhibition Increases IKAROS at *MTOR* Promoter (a)** Human B-ALL cells were transduced to express shRNA for *CK2α* (sh*CSNK2A1)* or scramble shRNA control (shCTL). Decreased transcription of CK2*α* (*CSNK2A1)* following transduction with sh*CSNK2A1* or control shCTL was analyzed by qRT-PCR. **(b)** qChIP analysis of IKAROS binding in control Nalm6 and 697 cells and following molecular CK2 inhibition with CK2*α* shRNA. Cells transduced with CK2*α* shRNA (sh*CSNK2A1)* show overall enhanced IKAROS binding to the upstream regulatory regions of the *MTOR* gene. *p<0.05, **p<0.01,

***p<0.001, ****p<0.0001.

**a**

**40 2 kb**


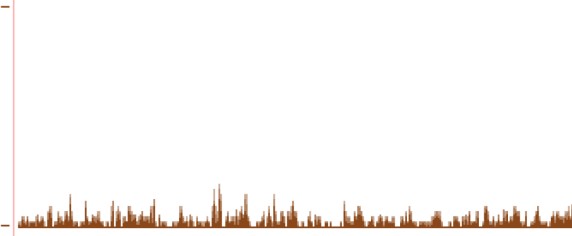

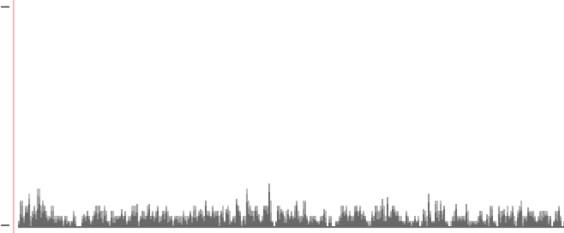


**Input**

### JM1 (CTRL)

**0 11,260,000 11,265,000 11,270,000**

**40 2 kb**

**IKAROS**

**0 11,260,000 11,265,000 11,270,000**

***MTOR***

**b**

**Chr1**

**Chr1**

**40 2 kb**

**Input**

### JM1 (CX-4945 treated)

**0**

**40 11,260,000 11,265,000 11,270,000**

**2 kb**

**IKAROS**

**0 11,260,000 11,265,000 11,270,000**

***MTOR***

**Chr1**

**Chr1**

**Supplemental Figure S5. Treatment with CK2 inhibitor enhances IKAROS binding at the *MTOR* promoter in human B-ALL cells. (a)** ChIP-seq binding of IKAROS in control JM1 cells and following CK2 inhibition with CX-4945. IKAROS binds the *MTOR* promoter with low affinity in control JM1 cells (which have high expression of CK2, data not shown). CK2 inhibition with CX-4945 strongly enhances IKAROS binding at the promoter of the *MTOR* gene. ChIP-seq data are analyzed with reference genome (MRCh38).


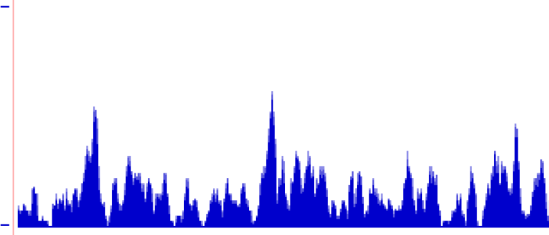

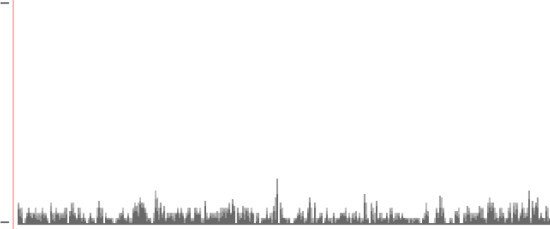


**a CX-4945 Increases IKAROS at**

***MTOR* Promoter in Ball Cell Lines**

**20**

********

********

********

********

*******

******

**Fold enrichment vs. IgG**

**15**

**10**


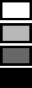


CTL–*α*-IgG CTL–*α*-IKAROS CX-4945–*α*-IgG

CX-4945–*α*-IKAROS

**5**

**0**

**Nalm6 697**

**b**

**CX-4945 Increases IKAROS at**

***MTOR* Promoter in Primary B-ALL**

**20**

********

********

********

********

********

********

********

********

********

**15**

**Fold enrichment vs. IgG**

**10**


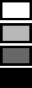


CTL–*α*-IgG CTL–*α*-IKZF1 CX-4945–*α*-IgG

CX-4945–*α*-IKAROS

**5**

**0**

**Patient 2**

**Patient 3**

**Patient 4**

**Supplemental Figure S6. CK2 regulates IKAROS binding at the *MTOR* promoter in human B-ALL cells (a)** qChIP analysis of IKAROS binding in control Nalm6 and 697 cells and following pharmacological CK2 inhibition with CX-4945. CK2 inhibition with CX-4945 strongly enhances IKAROS binding at the promoter of the *MTOR* gene. **(b)** qChIP analysis of IKAROS binding in control human B-ALL cells and following pharmacological CK2 inhibition with CX-4945. CK2 inhibition with CX-4945 strongly enhances IKAROS binding to the promoter of the MTOR gene. Patients 2 and 3 are Hispanic/Latino, patient 4 is non- Hispanic/Latino. Graphed data are the mean +/– SD of triplicates representative of one of 3 independent experiments. *p<0.05, **p<0.01, ***p<0.001, ****p<0.0001.

**a TBB Decreases *MTOR***

**Transcription**

**1.5**

********

********

CTL TBB

**Relative *MTOR* transcription**

**1.0**

**0.5**

**0.0**

**Nalm6**

**697**

**b TBB Increases IKAROS Binding at the *MTOR* Promoter**


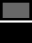


CTL–*α*-IgG CTL–*α*-IKAROS

TBB–*α*-IgG TBB–*α*-IKAROS

**15**

********

********

********

********

********

******

**Fold enrichment vs. IgG**

**10**

**5**

**0**

**Nalm6 697**

**Supplemental Figure S7. Effect of the CK2 inhibitor, TBB, on *MTOR* mRNA level. (a)** Nalm6 and 697 B- ALL cells were treated with TBB and *MTOR* mRNA levels were assessed by qRT-PCR; **(b)** qChIP analysis of IKAROS binding at the *MTOR* promoter in Nalm6 and 697 B-ALL cells treated with TBB. Cells were treated with 50μM TBB for 2 days. Graphed data are the mean +/– SD of triplicates representative of one of 3 independent experiments. *p<0.05, **p<0.01, ***p<0.001, ****p<0.000 1.

1. **CX-4945 Increases H3K27me3 at the *MTOR* Promoter**


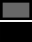


CTL–*α*-IgG CTL–*α*-H3K27me3

CX-4945–*α*-IgG

CX-4945–*α*-H3K27me3

**10**

**8**

*******

*******

********

********

*****

**ns**

**Fold enrichment vs. IgG**

**6**

**4**

**2**

**0**

**Nalm6 697**

1. **CX-4945 Decreases H3K9ac at the *MTOR* Promoter**


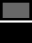


CTL–*α*-IgG CTL–*α*-H3K9ac

CX-4945–*α*-IgG

CX-4945–*α*-H3K9ac

**10**

********

*******

********

*******

**ns**

******

**Fold enrichment vs. IgG**

**8**

**6**

**4**

**2**

**0**

**Nalm6 697**

**Supplemental Figure S8. Effect of the CK2 inhibitor, CX-4945, on H3K27me^3^ and H3K9ac at the *MTOR* promoter**. **(a-c)** qChIP data show enrichment for **(a)** H3K27me^3^ and **(b)** loss of H3K9ac at the *MTOR* promoter following CX-4945 treatment versus non-treatment control (CTL) in Nalm6 and 697 B-ALL cells. Cells were treated with 10μM CX-4945 for 2 days. Graphed data are the mean +/– SD of triplicates representative of one of 3 independent experiments. *p<0.05, **p<0.01, ***p<0.001, ****p<0.000 1.

**CK2*α* Overexpression Decreases IKAROS at *MTOR* Promoter**

**4**

********

********

********

********

**ns**

**ns**

**Fold enrichment vs. IgG**

**3**


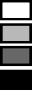


CTL–*α*-IgG CTL–*α*-IKZF1 *CSNK2A1*–*α*-IgG

*CSNK2A1*–*α*-IKAROS

**2**

**1**

**0 Nalm6**

**697**

**Supplemental Figure S9. CK2 overexpression inhibits IKAROS binding at the *MTOR* promoter in human B-ALL cells.** qChIP analysis of IKAROS binding in control Nalm6 and 697 cells and following CK2*α* overexpression. Overexpression of CK2*α* results in the loss of IKAROS binding to the upstream regulatory regions of the *MTOR* gene. Graphed data are the mean +/– SD of triplicates representative of one of 3 independent experiments. *p<0.05, **p<0.01, ***p<0.001, ****p<0.0001.

#### Patient 1 (W0)–Derived Xenograft Mice

**BM**

### a


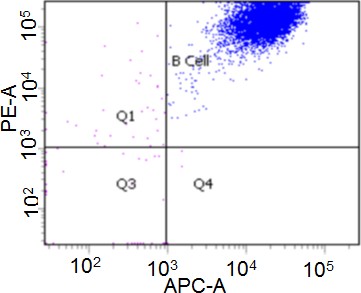

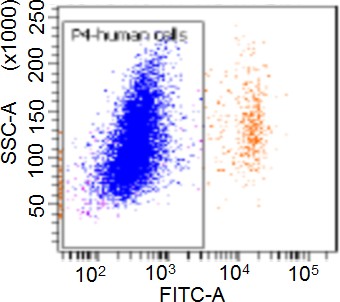


96.9%

Mouse CD45

**Control**

**Spleen**

98.5%


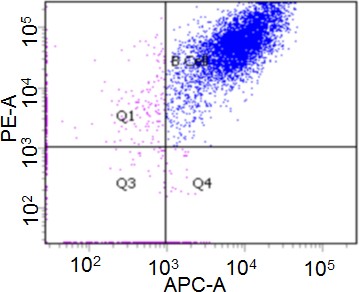


86.8%

Human CD19

Human CD10

Human CD10

Human CD19

### b


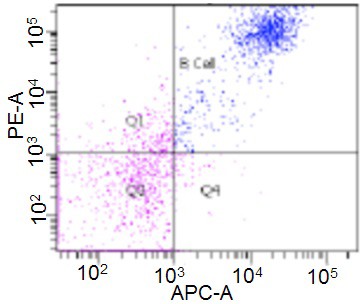

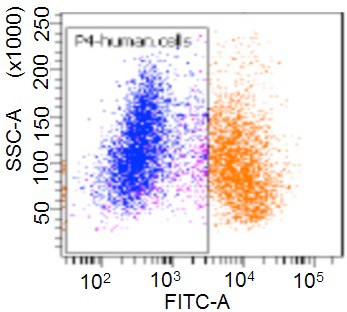


62.5%

Mouse CD45

**CX-4945 only**

91.8%

Mouse CD45

54.1%


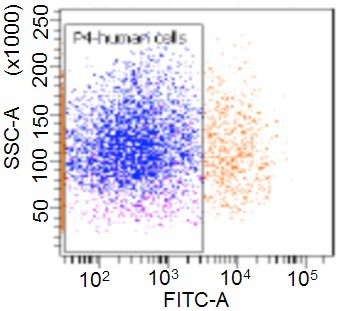

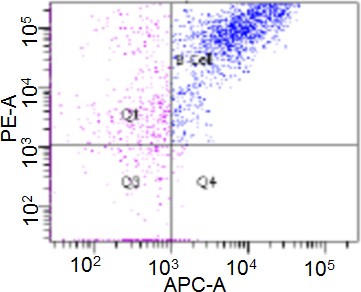


60.4%

Human CD19


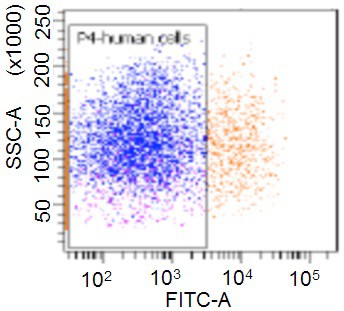


Human CD10

Human CD10

Human CD19

### Rapamycin only


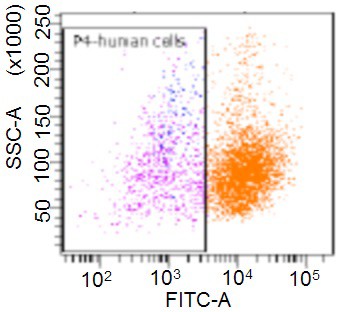


22.5%

Mouse CD45


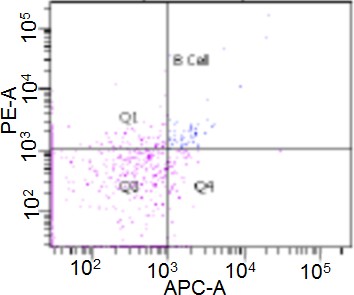


20.6%

Human CD19


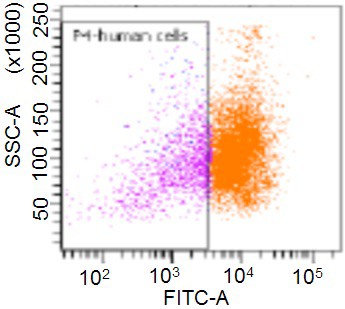


20.5%

Mouse CD45

Human CD10

Human CD10

56.1%

Mouse CD45

### CX-4945 + Rapamycin


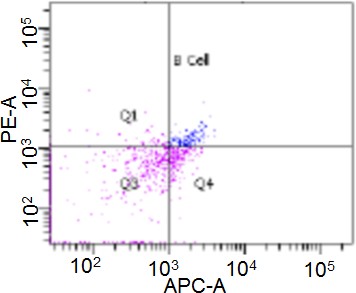


9.2%

Human CD19


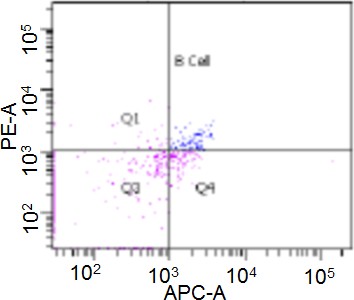


1.0%

Human CD19


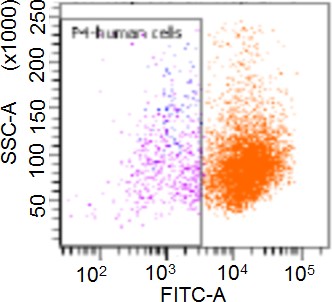


9.0%

Mouse CD45


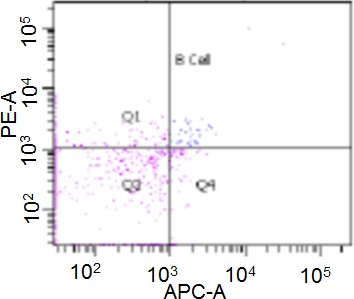


1.5%

Human CD19


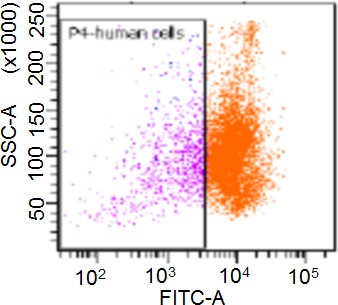


15.1%

Mouse CD45

Human CD10

Human CD10

**Supplemental Fig 10. Synergistic therapeutic effects of CX-4945 with rapamycin against B- ALL in in Patient 1-derived xenograft mice**. NRG mice were transplanted *via* tail vein with primary B-ALL cells (2x10E6 cells/mouse) from patient 1. Mice were randomly divided into four groups and treated with vehicle control, CX-4945 only, rapamycin only, or CX-4945 plus rapamycin as described in methods. **a-d:** Bone marrow and spleen cells were harvested and stained for flow cytometry to detect human B cell markers (CD10 and CD19), mouse CD45, and 7-AAD as a marker of dead cells. Plots shown were gated on total living (7-AAD–) cells and show mouse CD45– gates that were used to determine the percentage of engrafted human cells among total living cells (P4 in the right panels for BM and spleen columns). The plots are gated on total living mouse CD45– cells (left panels) and show the percentage of leukemia cells among the engrafted human cells based on the percentage of cells that were human CD19 and human CD10 double positive (right panels). Plotted are representative data from bone marrow (left column) and spleen (right column) in the vehicle control group (a panels) and the treatment group (b-c panels) of patient-derived xenograft mice transplanted with leukemia from patient 1, a Hispanic-Latino patient.

#### Patient 2 (W13)–Derived Xenograft Mice

**BM**

### a


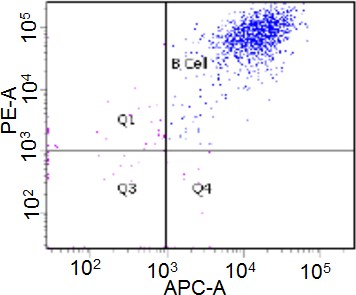

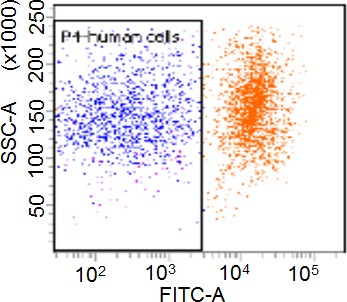


28.7%

Mouse CD45

**Control**

### Spleen

93.7%


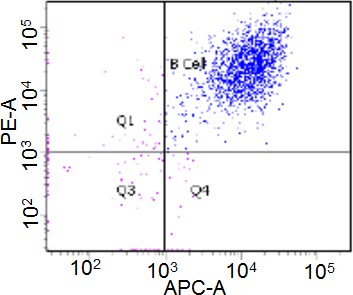


92.5%

Human CD19


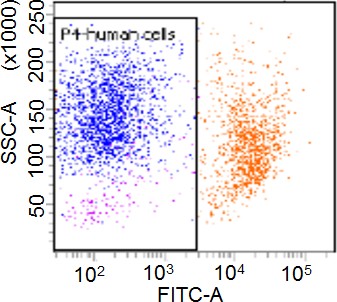


Human CD10

Human CD10

Human CD19

### b


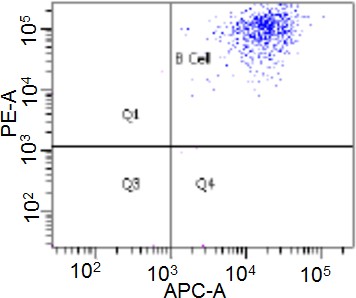

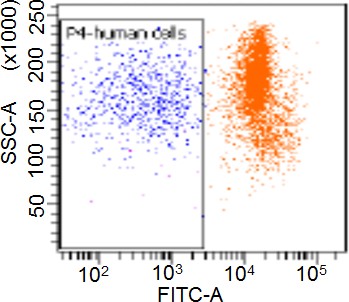


12.0%

Mouse CD45

**CX-4945 only**

45.0%

Mouse CD45

91.4%


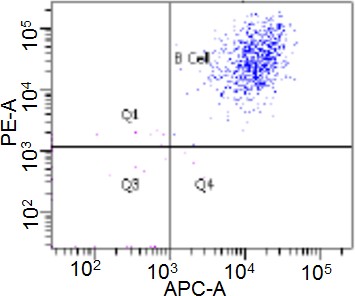


88.4%

Human CD19


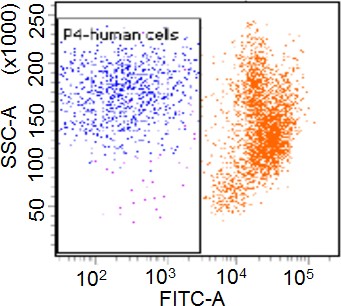


Human CD10

Human CD10

17.6%

Human CD19

### c


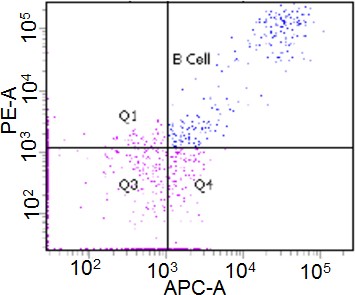


23.2%

Human CD19


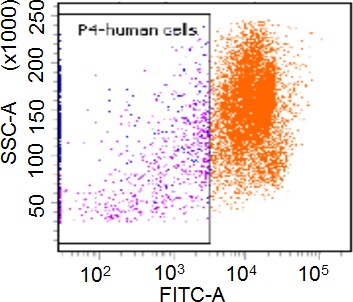


11.3%

Mouse CD45

Human CD10

**Rapamycin only**

Mouse CD45

### d CX-4945 + Rapamycin


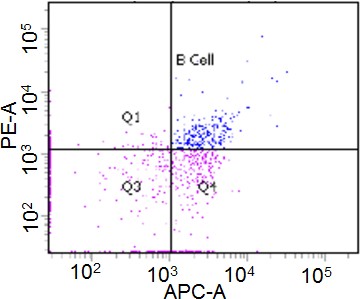


26.0%

Human CD19


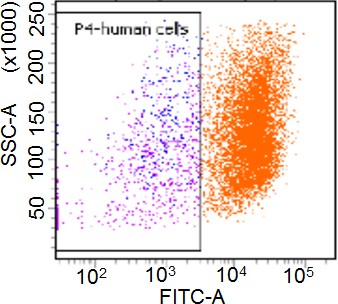


10.9%

Mouse CD45


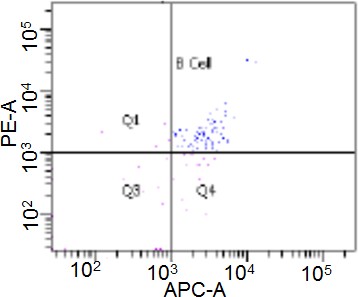


7.5%

Human CD19


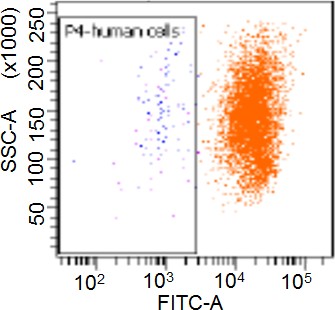


2.1%

Mouse CD45


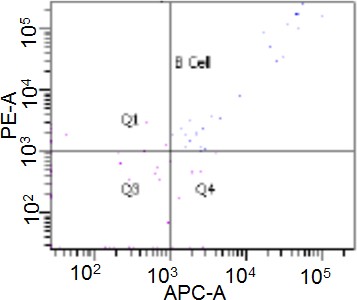


10.5%

Human CD19


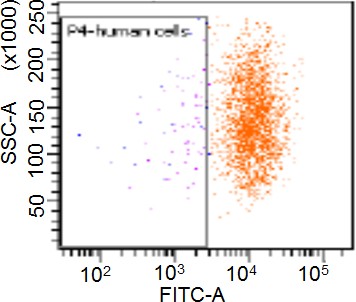


6.5%

Mouse CD45

Human CD10

Human CD10

Human CD10

**Supplemental Fig 11. Synergistic therapeutic effects of CX-4945 with rapamycin against B-ALL in Patient 2-derived xenograft mice**. NRG mice were transplanted *via* tail vein with primary B-ALL cells (2x10E6 cells/mouse) from patient 2. Mice were randomly divided into four groups and treated with vehicle control, CX-4945 only, rapamycin only, or CX-4945 plus rapamycin as described in methods. **a-d:** Bone marrow and spleen cells were harvested and stained for flow cytometry to detect human B cell markers (CD10 and CD19), mouse CD45, and 7-AAD as a marker of dead cells. Plots shown were gated on total living (7-AAD–) cells and show mouse CD45– gates that were used to determine the percentage of engrafted human cells among total living cells (P4 in the right panels for BM and spleen columns). The plots are gated on total living mouse CD45– cells (left panels) and show the percentage of leukemia cells among the engrafted human cells based on the percentage of cells that were human CD19 and human CD10 double positive (right panels). Plotted are representative data from bone marrow (left column) and spleen (right column) in the vehicle control group (a panels) and the treatment group (b-c panels) of patient-derived xenograft mice transplanted with leukemia from patient 2, a Hispanic-Latino patient.

#### Patient 3 (W31)-Derived Xenograft Mice

- 1. **BM**

### Control

**Spleen**


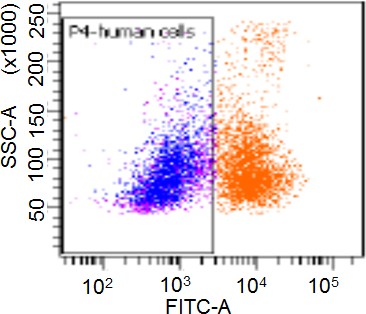
67.5%


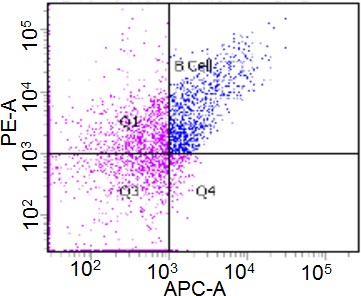


33.3%

Human CD19


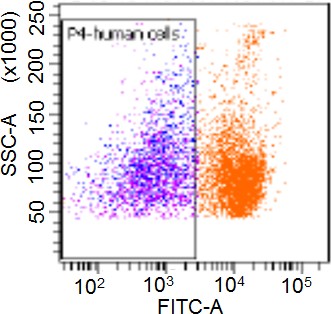


44.1%

**only**

Mouse CD45


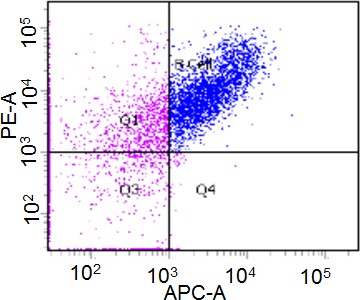


Human CD10

Human CD10

56.9%

- 1. Mouse CD45 Human CD19


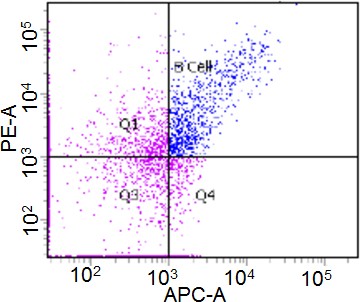


21.3%

Human CD19


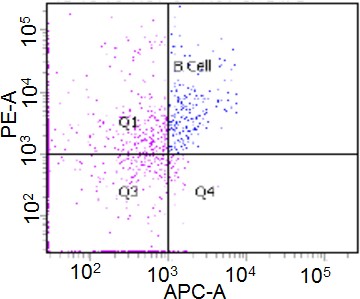


### CX-4945

35.1%


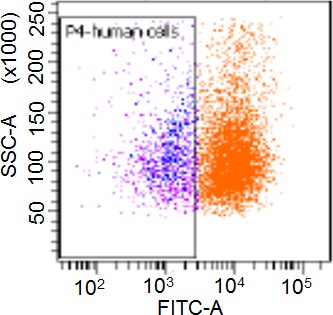

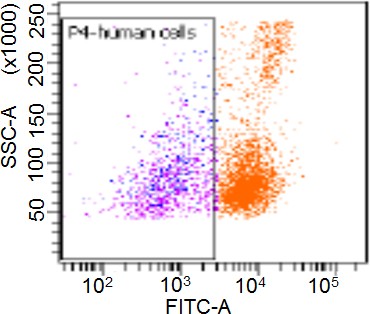


34.0%

Mouse CD45

Human CD10

Human CD10

16.0%

Human CD19

**c**

### Rapamycin only


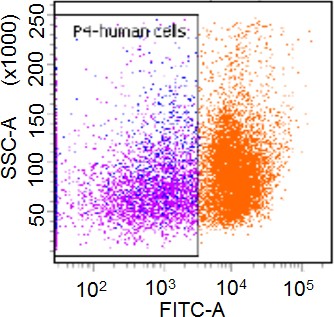


31.2%

Mouse CD45

Human CD10

Human CD10

Mouse CD45


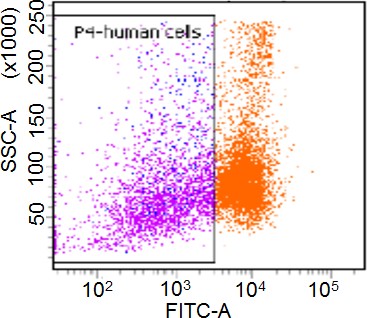


35.9%

Mouse CD45

### d CX-4945 + Rapamycin


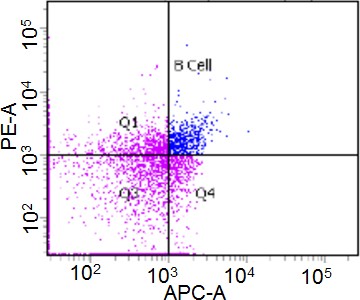


10.8%

Human CD19


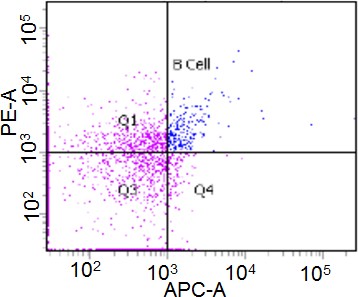


8.5%

Human CD19


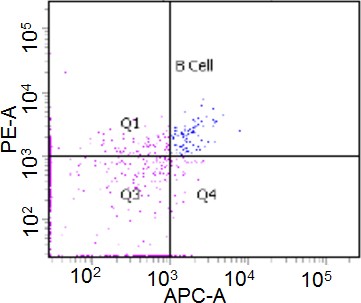


10.0%

Human CD19


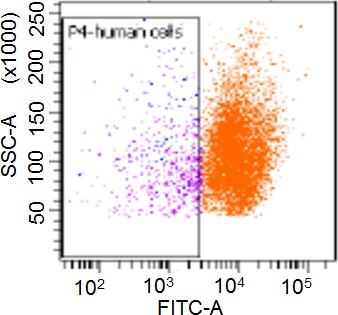


10.5%

Mouse CD45


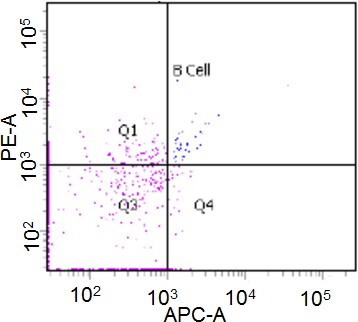


4.0%

Human CD19


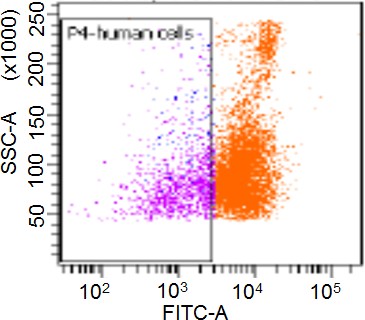


16.0%

Mouse CD45

Human CD10

Human CD10

**Supplemental Fig 12. Synergistic therapeutic effects of CX-4945 with rapamycin against B- ALL in in Patient 3-derived xenograft mice**. NRG mice were transplanted *via* tail vein with primary B-ALL cells (2x10E6 cells/mouse) from patient 3. Mice were randomly divided into four groups and treated with vehicle control, CX-4945 only, rapamycin only, or CX-4945 plus rapamycin as described in methods. **a-d:** Bone marrow and spleen cells were harvested and stained for flow cytometry to detect human B cell markers (CD10 and CD19), mouse CD45, and 7-AAD as a marker of dead cells. Plots shown were gated on total living (7-AAD–) cells and show mouse CD45– gates that were used to determine the percentage of engrafted human cells among total living cells (P4 in the right panels for BM and spleen columns). The plots are gated on total living mouse CD45– cells (left panels) and show the percentage of leukemia cells among the engrafted human cells based on the percentage of cells that were human CD19 and human CD10 double positive (right panels). Plotted are representative data from bone marrow (left column) and spleen (right column) in the vehicle control group (a panels) and the treatment group (b-c panels) of patient-derived xenograft mice transplanted with leukemia from patient 3, a Hispanic-Latino patient.

**a. Effect of CX-4945 on MTOR mRNA levels in PDX**

**1.0**

********

********

********

**Relative MTOR expression in PDX tissues**

**0.8**


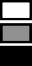


Control

CX-4945 Spleen

CX-4945 Bone Marrow

**0.6**

**0.4**

**0.2**

**0.0**

#### Patient 1 Patient 2 Patient 3

**b. Effect of CX-4945 on mTOR activity in PDX**

**
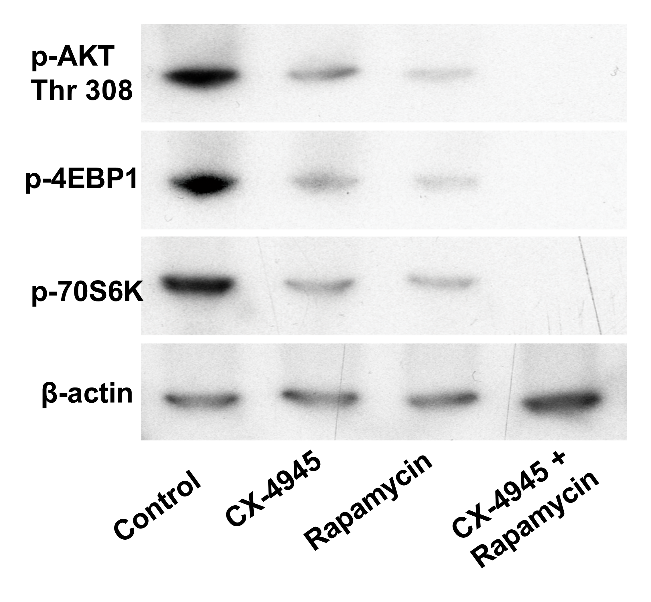
**

**Supplemental Figure S13. a: *MTOR* mRNA levels in bone marrow and spleen of PDX mice treated with CX-4945** Comparison of the relative *MTOR* expression levels as measured by qRT- PCR. Graphed data are the mean +/– SD of triplicate xenografts. Patients1, 2, and 3 are Hispanic- Latino. Statistical analysis by 2-way ANOVA followed by Dunnett’s multiple comparisons test,

****p<0.0001. **b:** **Rapamycin and CX-4945 treatment inhibit mTOR activity without inducing AKT activating loop.** Western Blot analysis of phosphorylated Akt form (Thr308), as well as of mTOR downstream targets (p-4EBP1 and phospho-p70S6K) in bone marrow leukemia cells from patient-derived xenograft of patient #1. Mice were treated with vehicle control, CX-4945 only, rapamycin only, or CX-4945 plus rapamycin as described in methods for 3 days.
